# Gastric Acid‐Responsive ROS Nanogenerators for Effective Treatment of *Helicobacter pylori* Infection without Disrupting Homeostasis of Intestinal Flora

**DOI:** 10.1002/advs.202206957

**Published:** 2023-05-01

**Authors:** Jiayin Yu, Zhihao Guo, Jiachang Yan, Changxin Bu, Chang Peng, Cuie Li, Rui Mao, Jian Zhang, Zhi Wang, Shi Chen, Meicun Yao, Zhiyong Xie, Chuan Yang, Yi Yan Yang, Peiyan Yuan, Xin Ding

**Affiliations:** ^1^ School of Pharmaceutical Science (Shenzhen) Shenzhen Campus of Sun Yat‐sen University Shenzhen 518107 P. R. China; ^2^ Guangzhou Institutes of Biomedicine and Health Chinese Academy of Science 190 Kaiyuan Avenue, Guangzhou Science Park, Luogang District Guangzhou 510080 P. R. China; ^3^ The Sixth Affiliated Hospital Sun Yat‐sen University Guangzhou 510655 P. R. China; ^4^ Bioprocessing Technology Institute (BTI) Agency for Science Technology and Research (A*STAR) 20 Biopolis Way, Centros #06‐01 Singapore 138668 Singapore; ^5^ Department of Orthopaedic Surgery Yong Loo Lin School of Medicine National University of Singapore Singapore 119288 Singapore

**Keywords:** chemodynamic therapy, gut microbiota, *Helicobacter pylori* infection, reactive oxygen species, sonodynamic therapy

## Abstract

*Helicobacter pylori* (*H. pylori*) has infected more than half of the world's population, and is the major cause of gastric cancer. The efficacy of standard antibiotic‐based triple therapy is declining due to drug resistance development. Herein, a pH‐responsive reactive oxygen species (ROS) nanogenerator (Fe‐HMME@DHA@MPN) composed of acid‐responsive metal polyphenol network (MPN) shell and mesoporous metal‐organic nanostructure core [Fe‐HMME (hematoporphyrin monomethyl ether, sonosensitizer)] loaded with dihydroartemisinin (DHA) is reported. These nanoparticles generate more ROS singlet oxygen than sonosensitizer HMME under ultrasonication, and this sonodynamic process is fueled by oxygen generated through Fenton/Fenton‐like reactions of the degraded product in gastric acid Fe (II) and hydrogen peroxide (H_2_O_2_) in the infection microenvironment. The encapsulated DHA, as a hydroperoxide source, is found to enhance the peroxidase‐like activity of the Fe‐HMME@DHA@MPN to generate ROS hydroxyl radical, beneficial for the microenvironment without sufficient H_2_O_2_. In vitro experiments demonstrate that the ROS nanogenerators are capable of killing multidrug‐resistant *H. pylori* and removing biofilm, and ROS nanogenerators show high therapeutic efficacy in a *H. pylori* infection mouse model. Unlike the triple therapy, the nanogenerators display negligible side effects toward the normal gut microbiota. Taken together, these self‐enhanced ROS nanogenerators have a great potential for treatment of *H. pylori* infection.

## Graphical abstract

A novel reactive oxygen species (ROS) nanogenerator, able to produce ROS in a self‐enhanced manner under gastric acidic condition through combination of sonodynamic therapy and chemodynamic therapy, has been prepared. These ROS nanogenerators can sterilize drug‐resistant *Helicobacter pylori* and treat infection in a mouse model, while avoiding side‐effects on gut microbiota, which are often caused by antibiotic‐based triple therapy.

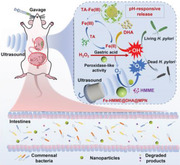

## 1 Introduction

*Helicobacter pylori* (*H. pylori*) is a spiral‐shaped microaerobic Gram‐negative bacterium that mainly colonizes in the human stomach and could cause various gastric diseases, such as gastritis, gastric ulcers, and gastric cancer.^[^ ^1^, ^2^, ^3^ ^]^ It is reported that over half of the world's population have been infected by *H. pylori* and such infections are the major cause of gastric cancer.^[^ ^4^, ^5^ ^]^ Triple therapy, including proton pump inhibitors and two antibiotics (usually amoxicillin or metronidazole and clarithromycin), is clinically used as the first‐line regimen for the treatment of *H. pylori* infection.^[^ ^6^, ^7^ ^]^ However, the degradation of antibiotics by gastric acid^[^ ^8^ ^]^ and the prevalence of antibiotic‐resistant strains reduce the therapeutic efficacy of the standard triple therapy.^[^ ^9^, ^10^ ^]^ In addition, antibiotic‐based treatments lead to undesired elimination of commensal bacteria which are closely related to human normal physiological processes, and the alteration of gut microbiota could result in a variety of diseases.^[^ ^11^, ^12^, ^13^, ^14^, ^15^ ^]^ Therefore, strategies that can effectively treat *H. pylori* infection without harming commensal bacteria are urgently needed.

Bacteria are highly susceptible to oxidative damages induced by exogenous reactive oxygen species (ROS) such as singlet oxygen (^1^O_2_) and hydroxyl radical (•OH).^[^ ^16^, ^17^, ^18^ ^]^ Importantly, the damage caused by ROS is selective, as mammalian cells are less susceptible to ROS than bacterial cells, presumably due to the ROS‐scavenging systems in mammalian cells can maintain the balance of ROS.^[^ ^19^, ^20^ ^]^ Sonodynamic therapy (SDT) is a promising non‐invasive therapeutic approach by using sonosensitizers to generate ^1^O_2_ to kill tumor cells or pathogenic microbes under ultrasonic conditions.^[^ ^21^ ^]^ Taking advantage of the high tissue penetration depth and safety of ultrasonic waves,^[^ ^22^ ^]^ SDT has been studied for treatment of various diseases^[^ ^23^, ^24^ ^]^ and several clinical trials for treatment of gliomas are undergoing (e.g., NCT04559685 at early phase 1 and NCT05123534 at phase 2). However, bacterial infection sites, particularly for the biofilm‐associated infections, are often hypoxic, and the lack of oxygen greatly limits the therapeutic efficacy of SDT, as oxygen is critical to produce ^1^O_2_ for SDT. Therefore, strategies that could increase the oxygen level or promote ROS generation in the infection site are needed for the application of SDT in the treatment of bacterial infections.

Apart from SDT, chemodynamic therapy (CDT) as another therapeutic approach using ROS to eliminate bacterial cells has recently attracted increasing attention. CDT is usually based on Fenton or Fenton‐like reaction which occurs between hydrogen peroxide (H_2_O_2_) and metal ions such as ferrous ions to generate •OH. Ferrous iron acting as a peroxidase can convert endogenous H_2_O_2_ at the site of infection to the highly reactive •OH,^[^ ^25^ ^]^ leading to the death of the pathogens. Moreover, the conversion of H_2_O_2_ to O_2_ by Fenton or Fenton‐like reactions has been leveraged to overcome hypoxia of tumor microenvironment.^[^ ^26^, ^27^, ^28^ ^]^ Hence, the enhanced SDT may be achieved if combined with CDT, as CDT can not only produce ROS but also supply oxygen to promote ^1^O_2_ generation of SDT. Although several nanosystems with combined SDT and CDT were reported to treat various tumors,^[^ ^22^, ^29^, ^30^, ^31^, ^32^ ^]^ the dynamic level of H_2_O_2_ varying from 65 µm to 88 mm ^[^ ^33^, ^34^, ^35^ ^]^ in the microenvironment of bacterial infection site limits the applications of SDT‐CDT combination therapy in the treatment of bacterial infections. Additional hydroperoxide sources may need to be incorporated in the SDT‐CDT nanosystems to enhance antibacterial activity regardless of the absence of sufficient H_2_O_2_.

**Scheme 1:**
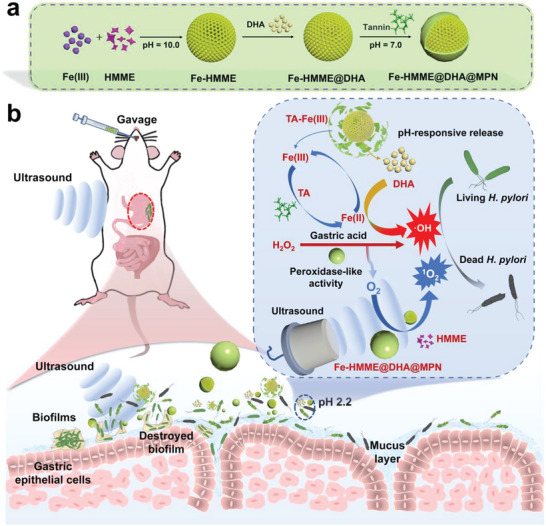
Schematic illustration of the functional mechanism of the ROS nanogenerator Fe‐HMME@DHA@MPN. a) Preparation of the nanogenerator. b) The therapeutic mechanism of the nanogenerator for the treatment of *H. pylori* infection through the synergy of sonodynamic therapy (SDT) and chemodynamic therapy (CDT) under ultrasound treatment. HMME: sonosensitizer hematoporphyrin monomethyl ether; DHA: anti‐malarial drug dihydroartemisinin; TA: polyphenol tannic acid.

Herein, we report a novel metal‐organic hybrid nanomaterial with pH‐responsive peroxidase‐like activity to achieve combined CDT and SDT for selective antibacterial capability toward *H. pylori* infection (**Scheme** **1**). The metal‐organic nanostructure (Fe‐HMME) composed of porphyrin‐related sonosensitizer hematoporphyrin monomethyl ether (HMME) and ferric iron through coordination was first prepared, followed by loading of an anti‐malarial drug dihydroartemisinin (DHA) that serves as hydroperoxide source for Fenton/Fenton‐like reaction.^[^ ^36^ ^]^ The polyphenol tannic acid (TA) was subsequently coated on the nanostructures, forming a metal polyphenol network (MPN) shell with Fe (III) to give the final ROS nanogenerator (Fe‐HMME@DHA@MPN). This MPN which is pH‐sensitive would dissociate under the gastric acidic pH,^[^ ^37^, ^38^ ^]^ and the release of Fe‐HMME and HMME in the gastric acid would be triggered by ultrasound (US) to produce ^1^O_2_ for SDT. Meanwhile, the dissociated TA would reduce Fe (III) to Fe (II), and the Fe (II) can catalyze H_2_O_2_ located at the microenvironment such as biofilm of *H. pylori* infection to generate •OH for CDT. Moreover, oxygen generated by the peroxidase‐catalysis of H_2_O_2_ would reduce the hypoxia influence and further boost SDT effect. At the H_2_O_2_‐deficient infection site, DHA released from the nanogenerator can provide hydroperoxide to react with Fe (II) through a Fenton‐like reaction to generate •OH for elimination of *H. pylori*. Furthermore, as the degraded products are biocompatible and the peroxidase‐like activity of the Fe‐HMME@DHA@MPN is suppressed in intestinal neutral conditions, minimal toxicity to normal intestinal flora is expected. Therefore, this cascade catalytic nanogenerator Fe‐HMME@DHA@MPN may be a promising synergistic SDT‐CDT strategy to combat *H. pylori* infection.

## 2 Results and Discussion

**Figure 1:**
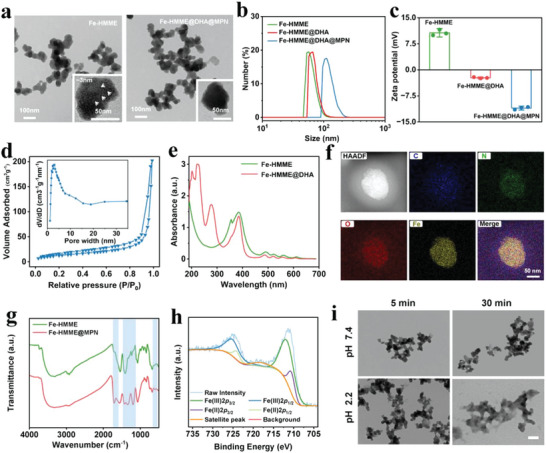
Characterizations of the catalytic ROS nanogenerator Fe‐HMME@DHA@MPN. a) TEM images of Fe‐HMME and Fe‐HMME@DHA@MPN nanostructures. Insets are the images of single nanoparticle (not the enlarged part of the image with low magnification), and white arrows represent mesoporous structure. b) Hydrodynamic sizes and c) zeta potentials of Fe‐HMME, Fe‐HMME@DHA, and Fe‐HMME@DHA@MPN nanoparticles. (*n* = 3, data are presented as mean ± SD) d) N_2_ absorption‐desorption isotherm and corresponding pore size distribution (inset) of Fe‐HMME. e) UV–vis absorption spectra of Fe‐HMME and Fe‐HMME@DHA nanoparticles. f) Element mapping of Fe‐HMME@DHA@MPN. g) FT‐IR spectra of Fe‐HMME and Fe‐HMME@MPN. h) XPS spectrum of Fe 2p in Fe‐HMME@MPN. i) TEM images of Fe‐HMME@MPN incubated in buffer solution at pH 7.4 or 2.2 for different periods of time (5 min and 30 min) (Scale bar: 200 nm).

The catalytic ROS nanogenerator Fe‐HMME@DHA@MPN was prepared through a three‐step procedure as shown in Scheme 1. Fe‐HMME nanostructure was first constructed via the coordination of HMME and Fe (III) using a simple one‐pot method. The resulting Fe‐HMME nanostructure exhibited a relatively uniform spherical morphology with an averaged diameter of ≈50 nm, and slightly positive charge (**Figure** **1**a–c and Figure S1, Supporting Information). Importantly, the Fe‐HMME possessed a well‐defined mesoporous structure with surface area of 64.3 m^2^ g^–1^, a pore volume of 0.32 cm^3^ g^–1^, and an average pore size of ≈3 nm (Figure 1d), which is beneficial for drug loading. DHA was loaded into the Fe‐HMME nanostructure through co‐incubation, and the characteristic peak at 290 nm in ultraviolet visible (UV–vis) spectroscopy confirmed the successful loading of DHA (Figure 1e). The loading content of DHA in Fe‐HMME was calculated to be 0.302 mg mg^–1^ (Figure S2, Supporting Information). The high DHA loading capacity was probably due to the electrostatic interaction between DHA and Fe‐HMME (Figure 1c), as well as the mesoporous structure of Fe‐HMME. The DHA‐loaded Fe‐HMME nanostructure was then coated with tannic acid (TA) through coordination with the Fe (III) in Fe‐HMME@DHA to form a polyphenol‐metal network shell, giving the Fe‐HMME@DHA@MPN. The coating of TA led to increased particle size (≈80 nm) and enhanced negative surface potential (−11.03 mV) (Figure 1a–c). The final product Fe‐HMME@DHA@MPN exhibited homogeneous structure revealed by the uniformly distributed C, N, O, and Fe elements shown in the images of high‐angle annular dark‐field scanning transmission electron microscopy (HAADF‐STEM) (Figure 1f), and the energy dispersive spectroscopy (EDS) line scan profiles of N, O and Fe elements within the single nanoparticle (Figure S3, Supporting Information). The formation of MPN in the tannic acid‐coated Fe‐HMME was verified by Fourier transform infrared (FT‐IR) spectroscopy (Figure 1g). Due to the coordination bond formed between polyphenol groups of TA with Fe (III) of Fe‐HMME, a part of the carboxylic groups originally bound to Fe (III) was exposed, resulting in the emergence of carboxylic stretching band at 1710 cm^–1^ (the free carboxylate band at 1427 cm^−1^). New absorption bands at around 1612 and 597 cm^−1^ ascribed to the C=O stretching of TA and the Fe‐O lattice vibrations, respectively, also confirmed the interaction between TA and Fe (III).^[^ ^39^, ^40^ ^]^

After the successful construction of Fe‐HMME@MPN, the redox status of Fe element that is critically important for CDT was analyzed by the X‐ray photoelectron spectroscopic analysis elemental survey (Figure S4, Supporting Information) and high‐resolution Fe 2p spectrum (Figure 1h). In the high‐resolution Fe 2p spectrum, the peaks of Fe (II) 2p_3/2_, Fe (III) 2p_3/2_, Fe (II) 2p_1/2_, Fe (III) 2p_1/2,_ and one satellite feature peak were observed. The redox status analysis of Fe in Fe‐HMME@MPN showed the ferrous Fe (II) peak accounted for 19.5% peak area (Figure 1h), while no ferrous Fe (II) was found for the Fe‐HMME (Figure S5, Supporting Information), indicating that ferrous Fe (II) was formed during the coordination reaction of gallate in TA with ferric Fe (III). The Fe (II) in the MPN structure endowed the nanoparticles with the reductive property, beneficial for the peroxidase‐like activity of nanoparticles that facilitate catalyzing the Fenton reaction. In addition to the reductive property, MPN coating also improved the stability of the Fe‐HMME. Fe‐HMME@MPN could be easily dispersed in water, and maintained good dispersity for 72 h, while the uncoated Fe‐HMME were not stable and quickly precipitated in 6 h (Figure S6, Supporting Information). The reductive property and enhanced stability further demonstrate the successful construction of Fe‐HMME@DHA@MPN.

As the coordination bond between Fe (III) and HMME or TA would dissociate under acidic conditions due to the breakage of Fe–O bond,^[^ ^41^ ^]^ Fe‐HMME@DHA@MPN nanoparticles would degrade in an acidic environment such as in stomach. In acidic solution (pH 2.2), the degradation of Fe‐HMME@DHA@MPN nanoparticles was indeed observed in 30 min (Figure 1i). Meanwhile, Fe‐HMME@DHA@MPN nanoparticles remained largely unchanged in PBS buffer (pH 7.4) for 30 min. The degradation kinetics of Fe‐HMME@DHA@MPN at different pH further demonstrated pH‐dependent degradation (Figure S7, Supporting Information). The degradation of Fe‐HMME@DHA@MPN under acidic conditions led to the release of DHA (Figure S8, Supporting Information), and 86.3% DHA was released at pH 2.2 in 120 min, while only 8.5% DHA was released at pH 7.4. MPN coating protected DHA from releasing at pH 7.4 and triggered the release of DHA under acidic conditions, which is desirable for the controlled release of DHA in stomach.

**Figure 2:**
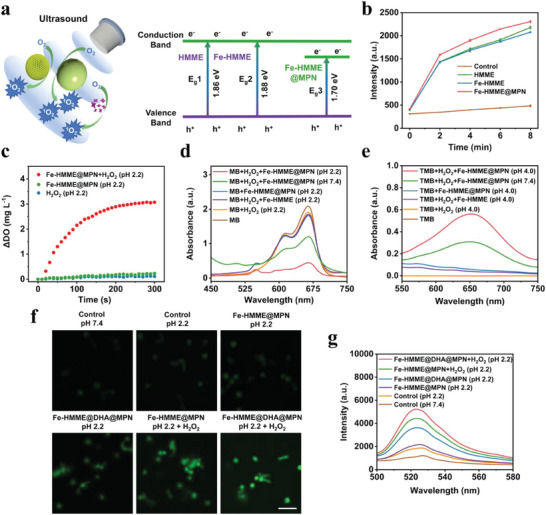
ROS production capability of the catalytic ROS nanogenerator. a) Schematic illustration of sonodynamic activity and energy bandgaps of HMME, Fe‐HMME, and Fe‐HMME@MPN measured by UV–vis diffuse reflectance spectra. b) The fluorescence spectra of the molecular probe singlet oxygen sensor green (SOSG) solution incubated with various samples (ultrasonication treatment time: 0 ‐ 8 min), showing Fe‐HMME@MPN generated the highest amount of ^1^O_2_. (*n* = 3, data are presented as mean ± SD). c) Time‐dependent O_2_ generation detected by an oxygen sensor. d) UV–vis absorption spectra of methylene blue (MB, 10 µg mL^–1^) incubated with various samples under different conditions (H_2_O_2_, 1 mm), suggesting the production of ·OH through the Fenton reaction of Fe‐HMME@MPN with H_2_O_2_ under acidic condition. e) UV–vis absorption spectra of the peroxidase substrate 3,3',5,5'‐tetramethylbenzidine (TMB, 120 µg mL^–1^) incubated with various samples under different conditions (H_2_O_2_, 1 mm), demonstrating peroxidase‐like activity of Fe‐HMME@MPN. f) Confocal images and g) fluorescence spectra of intracellular fluorescent ROS probe DCFH‐DA stained‐*H. pylori* (1 × 10^8^ CFU mL^–1^) treated with various samples under different conditions for 30 min. (scale bar: 5 µm). The concentrations of various samples used in (f,g) are as follows: Fe‐HMME@DHA@MPN, 120 µg mL^–1^; Fe‐HMME@MPN, 90 µg mL^–1^; H_2_O_2_, 100 µm.

After confirming the mesoporous structure, chemical composition, stability, and pH‐responsive cargo releasing of Fe‐HMME@DHA@MPN, the capability of ROS generation was analyzed. As HMME is an organic sonosensitizer that is able to react with O_2_ to produce ^1^O_2_ under ultrasonication, the energy bandgaps of HMME, Fe‐HMME, and Fe‐HMME@MPN, which reflect their capability of ROS generation under ultrasonication, were measured. Interestingly, Fe‐HMME@MPN exhibited a narrower bandgap of about 1.70 eV when compared with the bandgaps of HMME and Fe‐HMME at 1.86 eV and 1.88 eV, respectively (**Figure** **2**a and Figure S9, Supporting Information). The reduced bandgap of Fe‐HMME@MPN is conducive for electron‐hole separation under ultrasonication, and the resulting excited electron would react with O_2_ to produce ^1^O_2_.^[^ ^42^ ^]^ Therefore, Fe‐HMME@MPN should possess higher sonodynamic performance than HMME according to the bandgap theory. The ^1^O_2_ production characterized by the molecular probe singlet oxygen sensor green (SOSG) showed that Fe‐HMME@MPN produced the highest amount of ^1^O_2_ (Figure 2b), which was indeed in agreement with the energy band diagram (Figure 2a). In addition, the sonodynamic activity could be modulated by the ultrasonication duration (Figure S10, Supporting Information). The higher sonodynamic activity of Fe‐HMME@MPM could enable a better SDT effect even without the complete degradation of nanoparticles to release HMME, displaying a great potential of using catalytic Fe‐HMME@MPM as SDT sonosensitizer.

A limiting factor of ^1^O_2_ production via sono‐catalytic process in treating deep tissue infections, particularly for the biofilm‐related infections, could be the hypoxia microenvironment, as oxygen is indispensable for the generation of ^1^O_2_. One of the advantages of using Fe‐HMME@MPN nanoparticles as sonosensitizer is their peroxidase‐like property to produce oxygen in acidic environments. As shown in Figure 2c, Fe‐HMME@MPN produced a greater amount of oxygen in the gastric acid condition supplemented with H_2_O_2_ as compared to H_2_O_2_ or Fe‐HMME@MPN alone. The peroxidase‐like activity of Fe‐HMME@MPN is probably due to the Fenton reaction of Fe (II) with H_2_O_2_, which eventually produces oxygen.^[^ ^26^, ^27^, ^28^ ^]^ The generation of oxygen could alleviate the hypoxia influence in the infection site, enhancing sonodynamic efficacy. Under the simulated hypoxia condition, Fe‐HMME@MPN produced a higher amount of ^1^O_2_ than HMME and Fe‐HMME, demonstrating the importance of MPN formation on Fe‐HMME nanoparticles (Figure S11, Supporting Information). This is expected to enhance the sonodynamic therapeutic efficacy of the nanoparticles.

In addition to supplying oxygen to enhance sonodynamic activity, the Fe‐HMME@DHA@MPN nanoparticles were envisioned to possess chemodynamic activity, further boosting ROS generation through Fenton reaction under acidic conditions as depicted in Scheme 1. Fe‐HMME@DHA@MPN degrade under the acidic conditions to release encapsulated Fe (III) and TA, followed by the reduction of Fe (III) to Fe (II) by TA. Subsequently, Fe (II) would catalyze the H_2_O_2_ at the infection site to generate hydroxyl radical (•OH) through Fenton reaction. Moreover, another product of the Fenton reaction, Fe (III) with low catalytic activity would be again reduced to reactive Fe (II) mediated by TA, and continue catalyzing H_2_O_2_ to generate •OH (Scheme 1b). In order to verify this continuous and efficient Fenton reaction for CDT, the capability of Fe‐HMME@DHA@MPN to produce ·OH under the simulated gastric acid condition was analyzed. The absorbance of methylene blue (MB), the indicator of ·OH production, was notably diminished after incubation with Fe‐HMME@MPN and H_2_O_2_ under the acidic condition, suggesting the production of ·OH through the Fenton reaction (Figure S12, Supporting Information). The production of ·OH is concentration‐dependent, and an increased concentration of Fe‐HMME@DHA@MPN led to a higher amount of ·OH generated. Further MB experiments revealed that both low stomach pH and H_2_O_2_ were critical to ·OH production of the nanoparticles, as no significant generation of ·OH was observed for Fe‐HMME@MPN under the conditions with neutral pH or without addition of H_2_O_2_ (Figure 2d). The generation of ·OH by Fe‐HMME@MPN demonstrated a peroxidase‐like activity, which was further proven using the peroxidase substrate 3,3',5,5'‐tetramethylbenzidine (TMB). The peroxidase‐like activity of Fe‐HMME@MPN nanoparticles was both concentration and pH‐dependent (Figure 2e and Figure S13, Supporting Information), similar to other nanomaterials with peroxidase‐like activity.^[^ ^3^ ^]^ Notably, the intrinsic peroxidase‐like activity of Fe‐HMME@MPN at pH 7.4 was significantly weaker than that at pH 2.2 (gastric pH), which is desirable for treating *H. pylori* infection in stomach while being not harmful to the commensal intestinal microbes. The intracellular ROS generation in *H. pylori* characterized by confocal microscopy and fluorescence of ROS probe DCFH‐DA also confirmed the chemodynamic activity of Fe‐HMME@MPN under the acidic condition supplemented with H_2_O_2_ (Figure 2f,g).

Importantly, DHA loading in the Fe‐HMME@DHA@MPN nanoparticles led to the generation of ·OH (Figure S14, Supporting Information) and enhanced intracellular ROS even without the addition of H_2_O_2_ (Figure 2f,g), suggesting that DHA acted as a hydroperoxide source as the reactant of the Fenton/Fenton‐like reaction to generate ROS.^[^ ^36^, ^43^ ^]^ Therefore, the incorporation of DHA is important for Fe‐HMME@DHA@MPN to achieve potent chemodynamic activity particularly in the infectious microenvironment without sufficient H_2_O_2_. Although the pH‐responsive catalytic activity was previously reported to treat *H. pylori* infection,^[^ ^3^, ^44^ ^]^ we reported here for the first time a multifunctional nanocatalytic therapy that possesses both hydroperoxide self‐supply and SDT self‐enhancement properties for treatment of *H. pylori*. This approach could overcome the current limitations of the insufficient endogenous H_2_O_2_ in the complex infection microenvironment.

**Figure 3:**
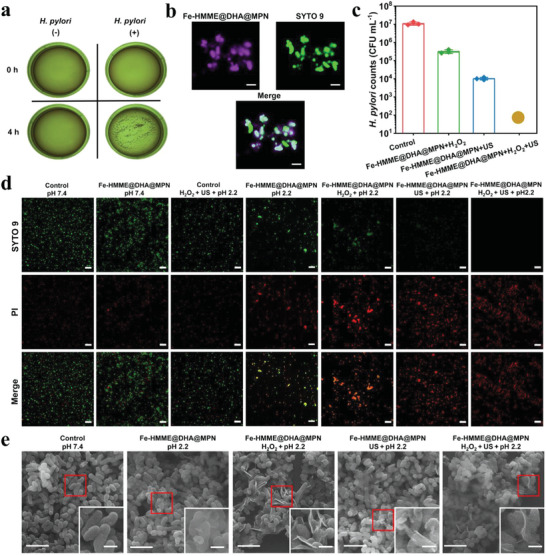
In vitro antibacterial activity of the catalytic ROS nanogenerator Fe‐HMME@DHA@MPN against *H. pylori*. a) The photographs of Fe‐HMME@DHA@MPN incubated with or without *H. pylori* in PBS. b) Confocal microscopic images of Fe‐HMME@DHA@MPN (purple fluorescence from HMME) incubated with *H. pylori* (incubation time: 30 min) stained with SYTO9 (green). c) Colony counts of *H. pylori* (initial count: 1 × 10^7^ CFU mL^–1^) after incubation with Fe‐HMME@DHA@MPN (40 µg mL^–1^) for 20 min under different conditions in the simulated gastric fluid (pH 2.2). The brown dot represents no bacterial colony observed (limit of detection: 100 CFU mL^–1^). (*n* = 3, data are presented as mean ± SD). d) Fluorescence images of *H. pylori* stained with live (green)/dead (red) staining kit after incubation with or without Fe‐HMME@DHA@MPN (40 µg mL^–1^) for 20 min under different conditions. Scale bar: 20 µm. e) SEM images of *H. pylori* treated with Fe‐HMME@DHA@MPN (40 µg mL^–1^) for 20 min under different conditions, and the insets are the magnified images of the selected areas. Scale bar: 3 µm (inset: 0.5 µm). US represents ultrasonication (US treatment time: 4 min).

Upon the confirmation of the ROS generation of Fe‐HMME@DHA@MPN through sonodynamic and chemodynamic processes, the in vitro antibacterial activity of the nanoparticles was investigated. When Fe‐HMME@DHA@MPN nanoparticles were incubated with drug resistant *H. pylori* (ATCC 43504), the originally well‐dispersed nanoparticles and bacteria formed aggregates (**Figure** **3**a and Figure S15, Supporting Information). Further confocal microscopic images showed that the nanoparticles (purple fluorescence from HMME) overlapped with SYTO9‐stained bacteria (Figure 3b), indicating that the aggregates were formed due to the adhesion of nanoparticles to bacteria instead of the aggregation of nanoparticles or bacteria cells themselves. The adhesion of the Fe‐HMME@DHA@MPN to *H. pylori* could be associated with the polyphenol groups, which form covalent or non‐covalent bonds such as hydrogen bond, van der Waals force or *π*‐*π* stacking force with the bacterial membrane.^[^ ^45^ ^]^ The aggregation of nanoparticles on *H. pylori* would reduce the travel distance of ROS generated by the nanoparticles, allowing for antibacterial efficacy as ROS has a short lifetime. The antibacterial activity of Fe‐HMME@DHA@MPN against *H. pylori* was evaluated, and the minimum bactericidal concentration (MBC) was tested to be >128.0 µg mL^–1^ in normal saline (0.9% m/m NaCl, pH 7.0) (Table S1, Supporting Information). When tested in the simulated gastric fluid (SGF, pH 2.2) supplemented with H_2_O_2_, Fe‐HMME@DHA@MPN nanoparticles at 40 µg mL^–1^ completely eliminated all the bacteria under ultrasonication (Figure 3c). Meanwhile, the nanoparticles without ultrasonication or H_2_O_2_ supplement displayed significantly reduced antibacterial activity. The combination of CDT and SDT enhanced the antibacterial activity of Fe‐HMME@DHA@MPN as more ROS were generated with both ultrasonication and H_2_O_2_ supplement (Figure 2). Importantly, Fe‐HMME@DHA@MPN with both sonodynamic and chemodynamic activity eradicated *H. pylori* in 20 min, while the conventional antibiotic amoxicillin did not exhibit significant killing efficiency even over a period of 40 min (Figure S16, Supporting Information).

In addition to the *H. pylori* resistant to the antibiotic metronidazole (ATCC 43504), a standard drug‐susceptible strain (ATCC 700392) and two clinically‐isolated antibiotic‐resistant strains (CS01 resistant to clarithromycin, LQ2# resistant to clarithromycin, amoxicillin, and levofloxacin) were also used to evaluate the bactericidal effect of Fe‐HMME@DHA@MPN. Strong bactericidal activity of HMME@DHA@MPN with low MBC at 5 – 10 µg mL^–1^ (Table S1, Supporting Information) and high killing efficacy >99.99% (Figure S17, Supporting Information) against various strains were observed in the presence of H_2_O_2_ under acidic condition. The excellent bactericidal ability against all the tested bacterial strains suggested that the nanogenerators Fe‐HMME@DHA@MPN are promising for the treatment of *H. pylori*.

The fluorescence‐based live/dead bacterial viability assays were performed to further analyze the antibacterial effect of Fe‐HMME@DHA@MPN (Figure 3d). At gastric pH (pH 2.2), the ratio of dead to live *H. pylori* cells significantly increased upon treatment of Fe‐HMME@DHA@MPN without addition of H_2_O_2_, indicating that the ROS generated by DHA‐Fe (II) reaction could lead to bactericidal effect. More dead cells were observed when added with H_2_O_2_ or treated with ultrasonication due to the enhanced ROS generation through chemodynamic or sonodynamic activity. Almost no live bacteria were found in the *H. pylori* sample treated with Fe‐HMME@DHA@MPN supplemented with H_2_O_2_ and ultrasonication under the same acidic condition, demonstrating the synergistic antibacterial activity of CDT and SDT. The fluorescence intensity of propidium iodide in bacteria under different treatment conditions revealed that ROS generated from Fe‐HMME@DHA@MPN damaged bacterial cell membrane and enhanced the permeability of the membrane (Figure S18, Supporting Information). The wrinkles and ruptured surface of bacterial cells observed from the scanning electron microscopic (SEM) images of Fe‐HMME@DHA@MPN‐treated bacteria (Figure 3e) also confirmed the cell wall/membrane damage of the treated bacteria. The damage of bacterial cell wall/membrane further resulted in protein leakage and eventually bacterial cell death (Figure S19, Supporting Information). Fe‐HMME@DHA@MPN with the physical antibacterial mechanism would less likely induce resistance in bacteria when compared with most antibiotics with specific intracellular drug targets.^[^ ^46^ ^]^

**Figure 4:**
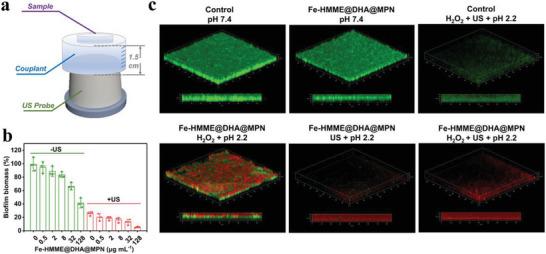
Anti‐biofilm activity of the catalytic ROS nanogenerator Fe‐HMME@DHA@MPN against *H. pylori*. a) Schematic diagram of the ultrasonic treatment. b) Eradication of mature *H. pylori* biofilms by treatment with Fe‐HMME@DHA@MPN at various concentrations for 20 min with or without ultrasonication characterized by biofilm biomass stained with crystal violet. (*n* = 3, data are presented as mean ± SD). c) Z‐stack images of *H. pylori* biofilm stained with live/dead staining kit after treatment with Fe‐HMME@DHA@MPN (128 µg mL^–1^) under different conditions for 20 min. US treatment time: 4 min.

Biofilm could protect the bacteria from the attacking of antibiotics, significantly reducing the susceptibility of bacteria to antibiotics.^[^ ^47^ ^]^ The eradication of *H. pylori* biofilm is a great challenge in treatment of *H. pylori* infections.^[^ ^48^ ^]^ Considering the excellent antibacterial activity of Fe‐HMME@DHA@MPN, anti‐biofilm activity of the nanoparticles was evaluated (**Figure** **4**a). The anti‐biofilm activity of Fe‐HMME@DHA@MPN was dose‐dependent, and 41.3% biomass reduction was achieved with a single 20 min treatment at 128 µg mL^–1^ (Figure 4b). Anti‐biofilm activity was enhanced with 94.5% biomass removed when ultrasonication was applied. The remarkable eradication efficiency was caused by the combination of the generated ROS and the physical damage by ultrasound. Notably, ultrasonication was capable of removing 75.4% biomass even in the absence of Fe‐HMME@DHA@MPN, suggesting the significant damage to the biofilm caused by ultrasonication. The z‐stack images of mature biofilm stained with live/dead viability kit after treatment of the nanoparticles were obtained to visualize the biofilm (Figure 4c). Ultrasonication alone could indeed remove the biofilm with fewer live bacteria (green fluorescence) left. However, only few red fluorescent dots were observed, suggesting that the treatment of ultrasound for 20 min physically disintegrated the biofilm instead of killing the bacteria in biofilm. In sharp contrast, the treatment of ultrasound combined with the Fe‐HMME@DHA@MPN resulted in lysis of bacteria in the biofilm as well as the disintegration of biofilm. The strong red fluorescence across the cross section observed implied that ROS generated through chemodynamic and sonodynamic processes penetrated across the biofilm that was disintegrated by the ultrasonication to kill the bacteria residing deep inside the biofilm. Therefore, the highest anti‐biofilm activity was achieved with the treatment of HMME@DHA@MPN under ultrasonication due to physical disintegration of biofilm by ultrasound, followed by the killing of the encapsulated bacteria by ROS generated through the sonodynamic and chemodynamic processes.

**Figure 5:**
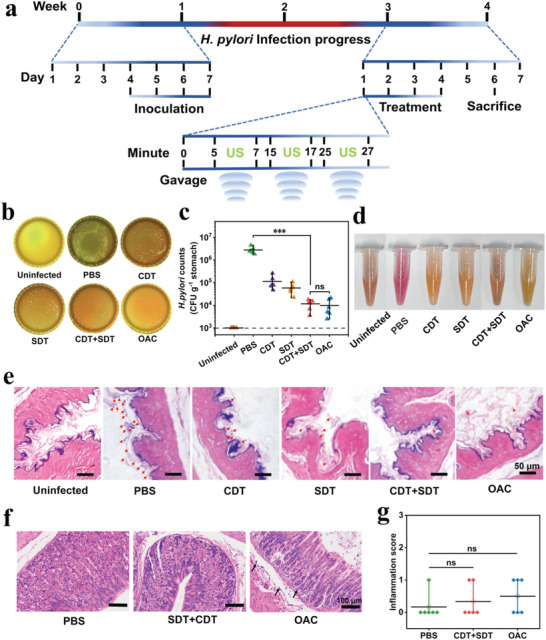
In vivo therapeutic effect of the catalytic ROS nanogenerator Fe‐HMME@DHA@MPN against *H. pylori* infection. a) Schematic diagram of *H. pylori* infection and treatment timeline in a BALB/c mouse model; b) Representative images and c) quantitative results of bacterial colonies in the stomach of *H. pylori*‐infected mice with different treatments (PBS: phosphate buffer saline, CDT: 30 mg kg^–1^ of Fe‐HMME@DHA@MPN, SDT: 16 mg kg^–1^ of Fe‐HMME + US, CDT+SDT: 30 mg kg^–1^ of Fe‐HMME@DHA@MPN + US, OAC: combination of 400 µmol kg^–1^ of omeprazole, 28.5 mg kg^–1^ of amoxicillin and 14.3 mg kg^–1^ of clarithromycin). The dot line represents the limit of detection. (*n* = 6). d) Urease test to evaluate *H. pylori* infection degree of the infected mice with different treatments. e) Gram staining of slices from the gastric mucosa of the mice with different treatments. Red arrows point to bacteria. f) H&E staining of slices from the gastric mucosa of the uninfected mice after treatment with PBS, CDT+SDT, or OAC. Black arrows represented inflammatory cell infiltration at the bottom of the mucosa and the submucosa. g) Inflammation score obtained by analyzing the H&E staining images in (f). Data are represented as means ± SD (*n* = 6), and the significant difference was analyzed by One‐way ANOVA with Tukey's post hoc test. *p < 0.05, **p < 0.01, ***p < 0.001 and ns representing non‐significance.

The therapeutic efficacy of Fe‐HMME@DHA@MPN was subsequently evaluated in the treatment of *H. pylori* infection in vivo. The *H. pylori* infection model was established using BALB/c mice. Each mouse was treated with 1 × 10^8^ CFU mL^−1^ *H. pylori* solution by gavage once a day for 4 consecutive days. Urease test, Gram‐staining of mouse gastric mucosa, and bacteria burden in the mouse stomach showed that the *H. pylori* infection in mice was successfully established 2 weeks post bacterial inoculation (Figure S20, Supporting Information). The *H. pylori*‐infected mice were randomly divided into five groups (*n* = 6), including PBS, CDT (Fe‐HMME@DHA@MPN), SDT (Fe‐HMME + US), CDT+SDT (Fe‐HMME@DHA@MPN + US), triple therapy OAC (omeprazole, amoxicillin, and clarithromycin), and one healthy mice group was used as control. The nanoparticles were then intragastrically administered to the *H. pylori*‐infected mice once a day for 4 days as displayed in **Figure** **5**a. For SDT and CDT+SDT groups, the mice were subjected to 2‐min ultrasound treatment for three times in 30 min. The bacteria detected in the stomach of the infected mice showed that the treatment of CDT+SDT had similar antibacterial efficacy as the OAC treatment with more than 99% CFU reduction compared with PBS‐treated mice (Figure 5b,c). In spite of notable bacterial reduction (>90%), CDT or SDT alone displayed weaker antibacterial activity than CDT+SDT and OAC treatment, demonstrating the in vivo synergistic antibacterial effect of CDT and SDT of Fe‐HMME@DHA@MPN. Moreover, the negative results of the urease test (Figure 5d), an important clinical test for *H. pylori*, also suggested the elimination of *H. pylori* in the stomach of CDT+SDT‐treated mice, which was similar to that of OAC‐treated mice. Gram staining of *H. pylori‐*infected gastric tissue further confirmed superior antibacterial activity of CDT+SDT (Figure 5e). Taken together, the combined CDT and SDT therapy mediated by Fe‐HMME@DHA@MPN effectively eliminated *H. pylori* in the stomach and demonstrated similar therapeutic efficacy as the standard triple therapy OAC in the treatment of *H. pylori* infection.

In addition to therapeutic efficacy against *H. pylori* infection, biocompatibility is critical for future clinical translation. Fe‐HMME@DHA@MPN showed no significant cytotoxicity toward GES‐1 (human gastric epithelial cell line) and HUVEC (human umbilical vein endothelial cell line) at concentrations up to 200 µg mL^–1^ with more than 80% cell viability (Figures S21 and S22, Supporting Information). Moreover, the degraded products of Fe‐HMME@DHA@MPN also displayed negligible cytotoxicity (Figure S21, Supporting Information), as these pH‐responsive nanoparticles can be degraded into small molecules with high biocompatibility under the gastric acidic condition. Even under the same ultrasound treatment as the antibacterial condition, the viability of gastric epithelial cells still reached 80%, suggesting that the ROS nanogenerator treatment under ultrasound induced minimal cytotoxicity (Figure S23, Supporting Information). The reduced cell viability is partially due to the use of ultrasound that could detach the cells from the culture plates. However, the condition for cell attachment in vivo is different from the in vitro culture in a plate. The in vivo toxicity to gastric epithelium would be evaluated in the following sections. Furthermore, Fe‐HMME@DHA@MPN nanoparticles were not toxic to typical commensal bacteria *Escherichia coli* (*E. coli*) and *Enterobacter aerogenes* (*E. aero*), which are important physiological flora in human or animal intestines (Figures S24 and S25, Supporting Information).^[^ ^49^ ^]^ The good biocompatibility of the nanoparticles is mainly due to the biocompatible components (the endogenous hemoglobin derivative HMME, necessary element Fe^3+^ and the green tea ingredient tannic acid, and these components are not toxic at pH 7.4. Fe‐HMME@DHA@MPN did not generate a significant amount of ROS at pH 7.4, and thus no obvious damage was seen to normal cells and the intestinal flora. These results suggested that Fe‐HMME@DHA@MPN possessed selective antibacterial activity against *H. pylori* and did not cause toxicity to normal cells and commensal bacteria.

The in vivo distribution and biosafety profiles of Fe‐HMME@DHA@MPN nanoparticles were further explored. The nanoparticles were mainly found in the gastrointestinal tract over 24 h after intragastric administration of a single dose, and no significant fluorescence of HMME was observed in major organs (heart, liver, spleen, lung, and kidney) (Figure S26a, Supporting Information). Importantly, the fluorescence from the free HMME in stomach started diminishing after 4 h, while that of Fe‐HMME@DHA@MPN in the stomach remained even after 24 h (Figure S26b, Supporting Information). This finding indicated that the nanoparticle formulation with MPN coating improved the retention time of sonosensitizer HMME, and the prolonged retention time could contribute to the enhanced CDT. The long retention time of the ROS nanogenerator Fe‐HMME@DHA@MPN could be ascribed to the adhesion property stemming from the polyphenol present on the surface of the nanoparticles.^[^ ^50^, ^51^, ^52^ ^]^ The H&E staining of gastric tissues in uninfected mice administered with Fe‐HMME@DHA@MPN showed no obvious inflammation or injury with a clear layer of epithelial cells and intact mucosa, which was similar to the gastric sample of mice treated with PBS (Figure 5f). Both inflammation score (Figure 5g) and injury score (Figure S27, Supporting Information) obtained by analyzing the H&E staining images also confirmed the biocompatibility of Fe‐HMME@DHA@MPN to the stomach with no significant difference as compared to the healthy mice treated with PBS (*p* > 0.05). The level of gastric proinflammatory cytokines was analyzed by enzyme‐linked immunosorbent assay (ELISA). As shown in Figure S28, Supporting Information, while the antibiotic (OAC) treatment led to a significantly increased level of the inflammatory cytokines, the treatment with ROS nanogenerators did not trigger significant gastric inflammation, which was consistent with H&E staining results. In addition, both H&E staining on the heart, liver, spleen, lung, and kidney of mice (Figure S29, Supporting Information) and the body weight of mice (Figure S30, Supporting Information) after CDT+SDT treatment demonstrated that oral administration with Fe‐HMME@DHA@MPN did not cause systematic toxicity. Moreover, the blood biochemistry analysis results also demonstrated that the repetitive usage of HMME@DHA@MPN with the same dosage as the treatment for *H. pylori* infections did not lead to systemic toxicity or Fe accumulation in blood (Table S2, Supporting Information). However, the OAC treatment caused a significant increase in the alanine aminotransferase (ALT) and aspartate amino transferase (AST) level, implying liver toxicity.

**Figure 6:**
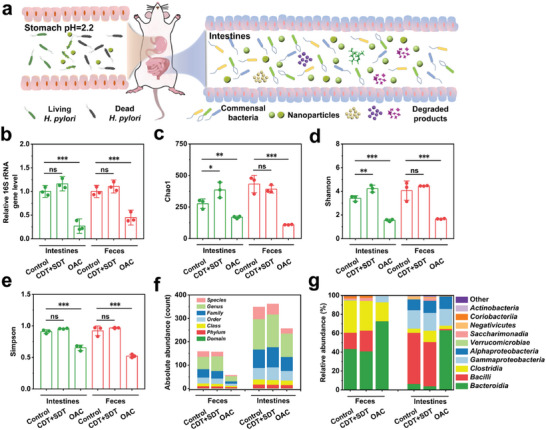
Influence of CDT+SDT therapy on the homeostasis of intestinal flora in comparison with the clinically used antibiotic‐based triple therapy (OAC). a) Schematic diagram of the selective sterilization of *H. pylori* in vivo using the gastric acid‐responsiveness of Fe‐HMME@DHA@MPN. Under the acidic stomach condition, Fe‐HMME@DHA@MPN catalyzed ROS production, showing significantly enhanced antibacterial efficacy. After entering the neutral intestinal environment (pH 7.4), the peroxidase‐like activity of Fe‐HMME@DHA@MPN was suppressed without showing a significant effect toward commensal bacteria. b) Effect of Fe‐HMME@DHA@MPN treatment on symbiotic bacteria in the intestines and feces of mice. Flora richness c) Chao 1 diversity, d) Shannon indexes, and e) Simpson indexes of *α*‐diversity in the intestines and feces of mice. f) Absolute abundance of microbiota in mouse feces and intestines determined by 16s rRNA sequencing test. g) Relative abundance of microbiota structure in mouse feces determined by 16s rRNA sequencing test. Data are presented as means ± SD (*n* = 3), and the significant difference was analyzed by One‐way ANOVA with Tukey's post hoc test. *p < 0.05, **p < 0.01, ***p < 0.001 and ns representing non‐significance.

In addition to their promising in vivo antibacterial efficacy in treating *H. pylori* infection, the catalytic ROS nanogenerator Fe‐HMME@DHA@MPN had negligible effects on the homeostasis of intestinal flora (**Figure** **6**a), as the nanoparticles and their degraded products are not toxic to commensal bacteria at pH 7.4 (Figures S24 and S25, Supporting Information). Fe‐HMME@DHA@MPN nanoparticles did not show bactericidal activity toward commensal bacteria in the gastrointestinal tract, while the standard OAC treatment significantly killed commensal bacteria with bacterial reduction in ileal contents and feces at 73.3% and 55.3%, respectively (Figure 6b). The richness (Chao1 index) and diversity (Shannon and Simpson indexes) of the ideal and fecal microbiota also confirmed the harmlessness of the catalytic ROS generator, while the microbiota ecology was largely altered by OAC treatment (Figure 6c–e). More specifically, the data of the absolute abundance at each taxonomic rank and microbial composition of the ideal and group and OAC treatment group, while the microbiome pattern of CDT+SDT treatment group resembled that of the untreated group (Figure 6f,g). Overall, the microbiome analysis results demonstrated that the balance of intestinal flora was largely unchanged with the treatment of the catalytic ROS nanogenerator, potentially mitigating the alternation of gut microbiota and avoiding various flora disorders caused by the antibiotic‐based triple therapy.

## 3 Conclusion

A gastric acid‐responsive ROS nanogenerator has been successfully made from biocompatible DHA, tannic acid, HMME, and Fe (II, III) to treat *H. pylori* infection in mice through sonodynamic and chemodynamic processes. The ROS nanogenerator catalyzed H_2_O_2_ in the infection microenvironment or DHA incorporated in the nanogenerator to continuously produce ROS in the acidic environment through Fenton/Fenton‐like reactions for CDT. The generation of ROS was further enhanced under ultrasonication fueled by oxygen generated by the Fenton reaction for SDT. The combination of CDT and SDT rapidly eliminated drug‐susceptible and drug‐resistant *H. pylori* and removed biofilm in a synergistic manner while not harming commensal bacteria and mammalian cells. The oral delivery of ROS nanogenerators eliminated *H. pylori* with potent in vivo therapeutic efficacy, which was comparable to that of the clinically‐used standard triply antibiotics therapy. More importantly, unlike the antibiotic‐based triple therapy, the treatment with the nanogenerators did not cause any side‐effects on the normal intestinal microflora in the intestines. These ROS nanogenerators provide a promising approach for the treatment of *H. pylori* infection.

## 4 Experimental Section

### Materials

Hematoporphyrin monomethyl ether (HMME) was obtained from Shanghai Yuanye Bio‐Technology Co., Ltd (China). Iron chlorides anhydrous (FeCl_3_), methanol, triethylamine (TEA), *N*,*N*‐dimethylformamide (DMF), dihydroartemisinin (DHA), tannic acid (TA), methylene blue (MB), TMB were purchased from Aladdin Bio‐Chem Technology Co., Ltd (Shanghai, China). Cell counting kit‐8 (CCK‐8) was bought from APExBIO (USA). Simulated gastric fluid (SGF, USP) was purchased from Leagene Biotechnology (China). Brain‐heart infusion (BHI) and Columbia blood plates were obtained from Huan kai Guangzhou Microbial (China). Singlet oxygen sensor green (SOSG) and live/dead BacLight bacterial viability kit (L‐7012) were bought from Invitrogen (USA). BCA protein assay kit was purchased from Beyotime (China). Mouse ELISA kits were purchased from NeoBioscience Technology Co, Ltd (China). *H. pylori* strain ATCC 43504 and ATCC 700392 were obtained from American Type Culture Collection (ATCC, USA). LQ2# and CS01 were clinical isolates gifted by Prof. Meicun Yao (Sun Yat‐sen University, Shenzhen, China.). BALB/c female mice (6–8 weeks old) were purchased from Gempharmatech Co., Ltd (China). High‐purify water (Millipore Milli‐Q grade) with a resistivity of 18.2 MΩ was used in all the experiments. All reagents used in the experiments were analytical grade without further purification.

### Instruments

The size distribution and zeta potential of the nanoparticles were measured on a Zeta Potential and Particle Size Analyzer (NanoBrook 90Plus PALS, Brookhaven, USA). TEM and elemental mapping images were obtained using transmission electron microscopy (FEI Tecnai G^2^ Spirit, Thermo Fisher Scientific, USA) with an accelerating voltage of 80 kV. Morphologies of the nanoparticles and cell surface were observed using a field‐emission scanning electron microscopy (SEM, JSM‐6330F, Japan). XPS (ESCALAB Xi+, Thermo Fisher Scientific, USA) was applied to analyze the valence state of elements. N_2_ adsorption/desorption and pore‐size distribution analysis were measured using a Quadrasorb 2MP apparatus (Quantachrome, USA). Automatic colony counter (Scan 300, Interscience, France) was applied for bacteria counting. Multifunctional microplate reader (Multiskan FC, Thermo Fisher Scientific, USA) was used to record the optical density (OD) of bacteria‐containing medium. Ultrasonic therapy instrument (Nu‐Tek, UT1021, China) was utilized to provide ultrasound (US) for SDT. UV–vis spectra were measured on a UV–vis spectrophotometer (T2602, Yoke Instrument, Shanghai, China). The band gap patterns were calculated from UV–vis diffuse reflectance spectra measured on UV–vis‐NIR spectrophotometer (Lambda 950, Perkin Elmer, USA). Fourier transform infrared spectra were measured on FT‐IR spectrophotometer (Nicolet6700‐Continuµm, Thermo Fisher Scientific, USA). Fluorescence spectra were recorded on a fluorescence spectrophotometer (FL970, Techcomp, Shanghai, China). Dissolved Oxygen Meter (JPSJ‐605F, Lei‐ci, Shanghai, China) was used to measure the amount of oxygen produced in solution. Confocal laser scanning microscopic (CLSM) imaging was performed using an LSM 880 NLO with a 63 × oil‐immersion objective lens (Zeiss, Germany).

### Preparation of Fe‐HMME@DHA@MPN Nanoparticles

HMME (5 mg) was dissolved in methanol/triethylamine solution (10 mL, v/v = 49:1), followed by the addition of methanol/DMF solution (10 mL, v/v = 85:15) containing FeCl_3_ (10 mg), and the mixture was under magnetic stirring for 2 h at room temperature. The mixture was ultrasonicated for 4 h in the dark by using an ultrasonic cleaning instrument (40 kHz) and then stirred overnight. Subsequently, Fe‐HMME nanoparticles were collected by centrifugation (15 000 rcf, 20 min) and washed with ethanol/DMF (5 mL, v/v = 85:15) three times.

The as‐synthesized Fe‐HMME nanoparticles (3 mg) were dispersed in ethanol (1.5 mL). Then DHA (3 mg) dissolving in ethanol/water solution (1 mL, v/v = 3:1) was added to Fe‐HMME nanoparticles suspension and magnetically stirred for 24 h at room temperature. Upon removal of the ethanol by evaporation, Fe‐HMME@DHA nanoparticles were obtained by centrifugation.

TA (40 mg) dissolved in water (1 mL) was added to Fe‐HMME@DHA nanoparticles aqueous suspension at Fe‐HMME@DHA of 0.4 mg mL^–1^ and TA of 0.4 mg mL^–1^. The suspension was vigorously mixed by a vortex mixer for 15 min. The colloidal particles were rinsed with Milli‐Q water by three centrifugation/redispersion cycles to remove excess TA and Fe (III). The resulting Fe‐HMME@DHA@MPN was re‐dispersed in 1 mL of Milli‐Q water.

### Stability of Fe‐HMME and Fe‐HMME@DHA@MPN Nanoparticles in Solutions

Fe‐HMME and Fe‐HMME@MPN at the concentration of 100 µg mL^–1^ were dispersed in Milli‐Q water. The stability of the nanoparticles was observed and photos were taken at different time intervals.

### pH‐Responsive Degradation of Fe‐HMME@DHA@MPN Nanoparticles

Fe‐HMME@DHA@MPN nanoparticles were re‐suspended in 1 mL of PBS (pH 7.4), Milli‐Q water (pH 7.0), or SGF (pH 2.2, 3, 4, and 5) to give a final concentration of 1.0 mg mL^–1^. After different incubation time intervals, the turbidity of different samples was measured by using the microplate reader, and the morphology of Fe‐HMME@DHA@MPN was observed under TEM.

### Determination of DHA Loading Content and In Vitro DHA Releasing

Fe‐HMME@DHA nanoparticles were washed with ethanol and Milli‐Q water for three times, respectively, to collect the free DHA in the supernatant. All ethanol and water in the washing procedures and the supernatant at the first centrifugation were collected to measure the loading content of DHA on a UV–vis spectrophotometer. Upon incubation with NaOH (0.2%) at 50 °C for 30 min, DHA was converted into a UV‐absorbing compound, which could be detected by the characteristic UV absorbance at 290 nm. The amount of DHA in the supernatant was calculated according to the standard absorbance‐concentration curve of DHA.

(1) $$\mathrm{The}\;\mathrm{DHA}\;\mathrm{loading}\;\mathrm{content}\;=\;\left(W_{\mathrm{loaded}\;\mathrm{DHA}}/W_{\mathrm{Fe}-\mathrm{HMME}@\mathrm{DHA}}\right)\;\times \;100\%$$

To study the release profile of DHA, Fe‐HMME@DHA@MPN (10 mg mL^–1^) nanoparticles were re‐dispersed in PBS (0.5 mL, pH 7.4) and hydrochloric acid solution (0.5 mL, pH = 2.2), respectively, followed by sealing in dialysis bags with a molecular weight cut‐off (MWCO) of 1000 Da and immersion in 10 mL of PBS or hydrochloric acid solution. The dialysis systems were incubated in a 37 °C shaker, and 1 mL of solution outside the dialysis bag was collected at different time intervals. After that, 1 mL of fresh PBS or hydrochloric acid solution was added back to the beaker. The concentration of released DHA was determined by using the UV–vis spectrophotometer.

### Detection of ·OH Generation

Fe‐HMME@MPN nanoparticles were dispersed in SGF at different pH (2.2 or 7.4) to give a concentration of 40 µg mL^–1^. After that, H_2_O_2_ (1 mm) and methylene blue (MB, 10 µg mL^–1^) were added to the mixture and incubated at 37 °C for 30 min. The absorbance of MB at 664 nm was monitored by using the UV–vis spectrophotometer. The generation of ·OH induced by Fe‐HMME@DHA@MPN or Fe‐HMME@MPN supplemented with H_2_O_2_ was detected using spin trap agent 5,5‐dietyl‐1‐pyrroline‐N‐oxide (DMPO) by electron spin resonance (ESR). In brief, Fe‐HMME@MPN (90 µg mL^−1^) supplemented with H_2_O_2_ (100 µm) or Fe‐HMME@DHA@MPN (120 µg mL^−1^) was added to pH 2.2 aqueous solution containing DMPO (5 mm) at 37 °C, which was then incubated for 0 or 10 min. The obtained supernatant was analyzed via ESR.

### Peroxidase‐Like Property of Fe‐HMME@DHA@MPN

The oxidation of TMB by Fe‐HMME @MPN nanoparticles and H_2_O_2_ in SGF (pH 4.0 or 7.4) produced a blue color with a major absorbance peak at 652 nm. Different combinations of TMB (final concentration: 120 µg mL^–1^), H_2_O_2_ (final concentration: 1 mm), and Fe‐HMME@DHA@MPN (final concentration: 40 µg mL^–1^) were added to SGF and incubated at 37 °C for 10 min, followed by measurement of UV–vis absorption to analyze the peroxidase‐like property of the nanoparticles.

### Detection of ^1^O_2_ Generation

To evaluate SDT function of Fe‐HMME@MPN, the Singlet Oxygen Sensor Green (SOSG) reagent was used for detecting ^1^O_2_ in aqueous solutions (pH 7.0 in normoxic group or 4.0 in hypoxia group). SOSG (10 µmol) and H_2_O_2_ (0 in normoxic group or 100 µm in hypoxia group) were added to the de‐ionized aqueous suspension of Fe‐HMME@MPN nanoparticles (1 mL, 30 µg mL^–1^). Among them, the liquid in the hypoxic group was boiled in advance and injected with argon to remove dissolved oxygen in the solution. Subsequently, the mixture was exposed to ultrasonication (US, 1.0 MHz, 70% duty cycle, 1.5 W cm^–2^) for 0, 2, 4, 6, 8 min, respectively. Fluorescence measurements were conducted on the spectrofluorometer using excitation/emission wavelength of 488/525 nm.

### Detection of O_2_ Generation

The dissolved O_2_ concentrations in SGF were measured by a JPSJ‐605F portable dissolved oxygen meter (Lei‐ci Instrument Co., Ltd, Shanghai, China). Briefly, Fe‐HMME@MPN nanoparticles were dispersed in SGF (pH 2.2) to give a concentration of 100 µg mL^–1^, followed by filling with argon, addition of a magnetic stir bar, and sealing with parafilm. Then the real‐time O_2_ concentrations were recorded under stirring after injection of H_2_O_2_ (1 mm). The O_2_ concentrations of control groups were measured by following a similar procedure.

### *H. pylori* Strain and Biofilm Cultivation

*H. pylori* ATCC 43504 was cryopreserved in BHI liquid medium containing 25% of glycerol at −80 °C. To obtain inoculum, *H. pylori* was cultured on a Columbia agar plate with 5% sterile defibrinated sheep blood and incubated under microaerobic conditions (5% O_2_, 10% CO_2_, 85% N_2_, and appropriate humidity) at 37 °C for 3–4 days. Mature *H. pylori* biofilms were formed by incubating bacterial suspension in BHI liquid medium supplemented with 5% fetal bovine serum (FBS) under microaerobic conditions (5% O_2_, 10% CO_2_, 85% N_2_) at 37 °C for 4–5 days.

### In Vitro *H. pylori* Adhesion Experiments

*H. pylori* (1× 10^7^ CFU mL^–1^) and Fe‐HMME@DHA@MPN (100 µg mL^–1^) were co‐incubated in PBS at 37 °C for 30 min, followed by observation of aggregated precipitates by CLSM. The fluorescence signal of Fe‐HMME@DHA@MPN from the intrinsic fluorescence of HMME was detected at *λ*_Ex/Em_ = 620/670 nm. SYTO9 staining was used to label live *H. pylori* for further exploration of the binding of Fe‐HMME@DHA@MPN and *H. pylori* (SYTO9: *λ*_Ex/Em_ = 488/501 nm).

### Antibacterial Assay against *H. pylori*

*H. pylori* were harvested by normal saline and adjusted to OD_600_ of 0.1. The as‐prepared bacterial suspension (100 µL) and a certain amount of Fe‐HMME@DHA@MPN were added to SGF (pH 2.2) supplied with 10 mm fresh urea, followed by incubation at 37 °C with shaking at 150 rpm for 30 min. The mixture was exposed to ultrasound (1.0 MHz, 70% duty cycle, 1.5 W cm^–2^) for 2 min during every 10 min of incubation. After that, the solution was placed on the Columbia agar plate by the spread plate method and cultured for 3–4 days before counting the number of the bacterial colonies. Based on the above measurements, the minimum concentration of nanoparticles that can kill ≥99.9% bacteria was defined as minimum bactericidal concentration (MBC).

### Killing Kinetics of Fe‐HMME@DHA@MPN against *H. pylori*

Fe‐HMME@DHA@MPN nanoparticles at different concentrations were dispersed in SGF at pH 2.2, followed by incubation with *H. pylori* at a concentration of 1 × 10^7^ CFU mL^–1^ in SGF supplied with fresh urea (10 mm) in the incubator. In the CDT treatment group, H_2_O_2_ (200 µm) was supplemented, whereas ultrasonication (1.0 MHz, 70% duty cycle, 1.5 W cm^–2^) for 2 min every 10 min during the 40 min incubation period was applied to the SDT treatment group. The suspension was then diluted and plated on Columbia agar plates, which were incubated for 72 h in the incubator before the bacterial colonies were counted.

### Evaluation of Biofilm Biomass

The anti‐biofilm activity of Fe‐HMME@DHA@MPN nanoparticles was evaluated by crystal violet assay. *H. pylori* suspension was incubated in a 96‐well microtiter plate to obtain mature biofilms according to the procedure described above. The obsolete medium was removed from biofilms, followed by the addition of fresh medium to wash and remove planktonic *H. pylori*. Subsequently, the culture plate was incubated with Fe‐HMME@DHA@MPN in pH 2.2 at different concentrations under the microaerobic conditions (5% O_2_, 10% CO_2_, 85% N_2_) at 37 °C for 20 min, and the mixture was exposed to ultrasonication (1.0 MHz, 70% duty cycle, 1.5 W cm^–2^) for 2 min during the incubation. At the end of incubation, the plate was washed with sterile PBS (pH 7.4) to remove suspension cells and stained with 0.1% (w/v) crystal violet for 30 min. After that, ethanol was added to dissolve crystal violet, and the biomass was measured by the absorbance of ethanol solutions at 570 nm (OD_570_) from the microplate reader.

### Live/Dead Fluorescence Staining

For planktonic *H. pylori*, after collection from Columbia blood plates, bacteria were dispersed in SGF (pH 2.2) (1 × 10^7^ CFU mL^–1^), followed by incubation with Fe‐HMME@DHA@MPN (40 µg mL^–1^) for 20 min under the microaerophilic atmosphere at 37 °C. Meanwhile, the bacteria without nanoparticles treatment were used as a control. The sample was centrifuged at 3500 rcf for 10 min to remove the supernatant. The residue was re‐suspended in 200 µL of normal saline and stained using the live/dead BacLight Bacterial Viability kits (Invitrogen, USA). Briefly, propidium iodide PI (100 µm) and SYTO9 (16.7 µm) were incubated with the suspension for 20 min, after which free dye was removed by centrifugation (3500 rcf for 10 min). The stained bacterial suspension was then loaded on a slide prior to CLSM imaging (SYTO9 for staining live cells, *λ*_Ex_: 488 nm, *λ*_Em_: 501 nm; propidium iodide (PI) for staining dead cells, *λ*_Ex_: 535 nm, *λ*_Em_: 617 nm).

For *H. pylori* biofilm cultivated for 5 days, Fe‐HMME@DHA@MPN nanoparticles (128 µg mL^–1^) were incubated with mature biofilms at 37 °C for 20 min. The remaining biofilms were stained with SYTO9 (16.7 µm) and PI (100 µm) in dark for 20 min and observed by using the CLSM.

### Cell Morphology Characterized by SEM

After 20 min incubation with Fe‐HMME@DHA@MPN nanoparticles in SGF at 37 °C, the bacteria were re‐dispersed in PBS and centrifuged at 3500 rcf to remove the supernatant. The bacteria were added to 2.5% glutaraldehyde and incubated overnight at 4 °C. The above bacterial suspension was then centrifuged (2000 rcf, 10 min) and dehydrated via treatment with 50%, 70%, 90%, and 100% gradient ethanol for 10 min, respectively. Finally, the samples were freeze‐dried and sputter‐coated with gold. The morphology of *H. pylori* was observed under SEM.

### Antibacterial Mechanism Study

*H. pylori* were dispersed in SGF (pH 2.2) (1 × 10^7^ CFU mL^–1^), followed by incubation with Fe‐HMME@DHA@MPN (40 µg mL^–1^) for 20 min under the microaerophilic atmosphere at 37 °C. The sample was centrifuged at 3500 rcf for 10 min to remove the supernatant. i) Propidium iodide (PI) staining of *H. pylori*. The residue was re‐suspended in 200 µL of normal saline and stained using the red dye PI (100 µm), and the fluorescence intensity was measured. ii) *H. pylori* protein leakage. The pH of the supernatant was adjusted to neutral and incubated with the test solution for 30 min, the amount of leaked proteins was determined by BCA protein assay kit.

### Cytotoxicity Test

Cell viability was evaluated with the cell counting kit‐8 (CCK‐8). Human gastric epithelial cells (GES‐1, 5 × 10^3^ cells per test well) and human umbilical vein endothelial cells (HUVEC, 5 × 10^3^ cells per well) were cultured in DMEM (Gibco) supplemented with 10% fetal bovine serum and 1% penicillin‐streptomycin on a 96‐well plate for 24 h at 37 °C. Subsequently, Fe‐HMME@DHA@MPN nanoparticles and their degradation products at different concentrations (0 ‐ 200 µg mL^–1^) were added to the cells and incubated for 24 h. The degradation products of Fe‐HMME@DHA@MPN nanoparticles were obtained by incubating the nanoparticles in SGF for 4 h at different concentrations (0 ‐ 200 µg mL^–1^), followed by adjustment of pH to neutral and lyophilization. The cells were washed with DMEM three times, and 10% CCK‐8 solution was added. After incubation in 5% CO_2_ at 37 °C for 2 h, the absorbance value at 450 nm was measured by using the microplate reader. In addition, cell viability of GES‐1 treated with Fe‐HMME@DHA@MPN under ultrasound was also evaluated. Briefly, GES‐1 cells (5 × 10^3^ cells per test well) were incubated with Fe‐HMME@DHA@MPN (40 µg mL^–1^) for 30 min, and treated with ultrasound (1.0 MHz, 70% duty cycle, 1.5 W cm^–2^) twice for 2 min each time. The CCK‐8 assay was performed as described aforementioned after incubating the treated cells for 24 h.

### In Vitro Antibacterial Activity of Fe‐HMME@DHA@MPN Nanoparticles against Intestinal Flora

*E. coli* and *E. aero* suspension were adjusted to an OD_600_ of 0.07 with normal saline. The as‐prepared bacterial suspension (1 × 10^7^ CFU mL^–1^) and a certain amount of Fe‐HMME@DHA@MPN (100 µg mL^–1^) were incubated in PBS (pH 7.4) at 37 °C for 6 h. The sample was then centrifuged at 3500 rcf for 10 min to remove the supernatant. The residue was stained with SYTO9 and PI in dark for 15 min and observed under CLSM. Meanwhile, the suspension was placed and spread on the Mueller‐Hinton agar plate and cultured for 24 h before counting the bacterial colonies.

### In Vivo Antibacterial Experiments

All the in vivo experiments performed were approved by the Institutional Animal Care and Use Committee of Guangzhou Institutes of Biomedicine and Health (GIBH), Chinese Academy of Science (Approval number: N2022135). BALB/c female mice (6–8 weeks old) were purchased from Gempharmatech Co., Ltd (China) and employed in accordance with the guidelines approved by the Laboratory Animal Welfare and Ethical Committee of GIBH. Each BALB/c mouse was treated by oral gavage with 200 µL of 1 × 10^8^ CFU mL^−1^ *H. pylori* in PBS once a day for 4 days (on days 4, 5, 6, and 7, respectively), and the infection progress was allowed to develop for 2 weeks. After that, the infected mice were euthanized, and the stomach tissue was removed along the greater curvature, followed by Gram staining,^[^ ^3^ ^]^ urease test,^[^ ^53^ ^]^ and bacterial plating. The colony forming unit (CFU) of *H. pylori* was normalized to the tissue weight (*n* = 6). The above experiments were used to demonstrate the successful colonization of *H. pylori* in the stomach of BALB/c mice, and this same infection model was used for the subsequent in vivo antibacterial experiments.

The *H. pylori*‐infected mice were randomly divided into 5 treatment groups (*n* = 6) to receive PBS, Fe‐HMME@DHA@MPN (CDT group), Fe‐HMME with ultrasonication (SDT group), Fe‐HMME@DHA@MPN with ultrasonication (30 mg kg^–1^, CDT+SDT group) or triple therapy OAC (400 µmol kg^–1^ of omeprazole, 28.5 mg kg^–1^ of amoxicillin, 14.3 mg kg^–1^ of clarithromycin) by oral gavage once a day for 4 consecutive days. The mice treated with PBS were used as negative controls. The dose of Fe‐HMME@MPN and Fe‐HMME groups was quantified based on 30 mg kg^–1^ of Fe‐HMME@DHA@MPN. For the triple therapy (OAC) group, mice were treated with proton pump inhibitor (omeprazole) for 30 min before intragastric administration of antibiotics to neutralize gastric acid and prevent potential antibiotic degradation. For the treatment groups that required ultrasonic irradiation, the stomach of the mice was sonicated three times in total 6 min during the 30 min treatment period using an ultrasonic therapy instrument (1.0 MHz, 70% duty cycle, 1.5 W cm^–2^) after each oral gavage, and the coupling agent was filled between the probe and the skin of the mice. The mice were sacrificed on the second day after all treatments, and the stomach tissues were collected for evaluation of treatment efficacy in each group by Gram staining, urease test, and colony plating. For *H. pylori* plating, gastric tissue was suspended in 1 mL of PBS and homogenized, followed by serial dilution and being spotted on Columbia agar plate with 5% sterile defibrinate sheep blood, and multiple antibiotics (10 µg mL^–1^ vancomycin, 5 µg mL^–1^ cefsulodin sodium, 5 µg mL^–1^ trimethoprim lactate, and 5 µg mL^–1^ amphotericin B). Subsequently, the plates were incubated at 37 °C under the microaerobic conditions (5% O_2_, 10% CO_2_, 85% N_2_) for 4 days, and in vivo antibacterial activity was evaluated by counting bacterial colonies.

### Histological Staining

The organ tissue samples fixed with formaldehyde were dehydrated and then embedded in paraffin. The sections of 5 µm thickness were obtained by using a slicing machine, which was used for Gram staining and H&E staining to evaluate colonization of *H. pylori*, inflammation, and injury in the stomach.

### Ex Vivo Biodistribution and Accumulation of Fe‐HMME@DHA@MPN Nanoparticles

Fe‐HMME@DHA@MPN nanoparticles (30 mg kg^–1^) and HMME (15 mg kg^–1^) were administrated by oral gavage. Major organs were harvested and imaged ex vivo by the in vivo fluorescence imaging system (IVIS Lumina III, PerkinElmer, USA) at 0.5, 1, 4, and 24 h post intragastric administration. Fe‐HMME@DHA@MPN nanoparticles were auto‐fluorescent due to the intrinsic fluorescence of HMME at *λ*_Ex/Em_ = 620/670 nm. Relative retention ratio of free HMME and Fe‐HMME@DHA@MPN in stomach harvested from mice upon receiving the aforementioned treatment was calculated by measuring the fluorescence intensity at the stomach in the reference to the fluorescence intensity at 0.5 h.

### In Vivo Biocompatibility of Fe‐HMME@DHA@MPN Nanoparticles

Mice were given a daily gavage of PBS, Fe‐HMME@DHA@MPN (30 mg kg^–1^), and OAC for 4 consecutive days. The stomach of the mice in Fe‐HMME@DHA@MPN treatment group was sonicated three times in total 6 min over the 30 min treatment period using an ultrasonic therapy instrument (1.0 MHz, 70% duty cycle, 1.5 W cm^–2^) after each oral gavage. Mice were sacrificed on day 6, and the stomach, heart, liver, spleen, lung, kidney, feces, and colonic segment of each mouse were collected. The stomach, heart, liver, spleen, lung, and kidney tissues were kept in formalin for H&E staining. The inflammation and injury of stomach were scored in a blind manner based on the H&E staining. The specific scoring criteria used were detailed using a method reported previously.^[^ ^6^ ^]^ Enzyme‐linked immunosorbent assay (ELISA) was also performed to analyze the inflammation. The gastric tissues obtained from each group were accurately weighed and homogenized. The homogenates were centrifuged at 5000 rpm for 10 min at 4 °C, and the levels of IL‐1*β*, IL‐6, and TNF‐*α* in the supernatant were measured using ELISA kits according to the manufacturer's instructions.

The bacterial DNA from the ileal content and feces was obtained by the Soil&Stool Genome DNA Extraction Kit (K3115‐S, Ige Biotechnology). Meanwhile, the integrity and concentration of the bacterial DNA were measured by agarose gel electrophoresis and nanodrop instrument (Thermo Fisher Scientific), respectively. The amplification of the V3‐V4 region (341F‐805R) of the 16S rRNA gene was conducted with universal primer pairs (341F 5′‐CCTACGGGNGGCWGCAG‐3′ and 805R 5′‐GACTACHVGGGTATCTAATCC‐3′) using the Ipure SYBR Green qPCR Master Mix (Ige Biotechnology) and the StepOnePlus (Applied Biosystems). The entire sequencing process was determined in Keyida Biological Technology Co., Ltd (China). The abundance, diversity, and structure of gut microbiota in the ileal content and feces were analyzed. The bacterial load was determined by quantitative real‐time PCR using a protocol modified from the reported method,^[^ ^54^, ^55^ ^]^ and the quantitative standard curve was made by pGSI (Ige Biotechnology) vector containing a nearly full length copy of the 16S rRNA gene *Porphyromonadaceae pasteri* strain KUFDS01. Moreover, the primer sequences for quantitative real‐time PCR were listed in Table S3, Supporting Information. The data of the bacterial load were normalized to the weight of ileal content and feces, respectively.

### Statistical Analysis

The significant difference between two groups was analyzed by Student's *t*‐test. The significant difference between multiple groups was analyzed by One‐way ANOVA with Tukey's post hoc test. All experiments were performed at least three times, and the results are presented as mean ± SD. Significance is indicated by ns (non‐significance), *p < 0.05, **p < 0.01, ***p < 0.001. All data were analyzed by Excel or GraphPad Prism 8.0.

## Conflict of Interest

The authors declare no conflict of interest.

## Supporting information

Supporting Information

## Data Availability

The data that support the findings of this study are available from the corresponding author upon reasonable request.
