# Supplementary material for: Gastric Acid‐Responsive ROS Nanogenerators for Effective Treatment of Helicobacter pylori Infection without Disrupting Homeostasis of Intestinal Flora
Source: Adv Sci (Weinh). 2023 May 1;10(20):2206957. doi: 10.1002/advs.202206957 (PMC10369278; doi:10.1002/advs.202206957)
Supplement: Supplementary file 1 — Supporting Information [file ADVS-10-2206957-s001.pdf]

ADVANCED SCIENCE
Open Access

Supporting Information

for *Adv. Sci.*, DOI 10.1002/advs.202206957

Gastric Acid-Responsive ROS Nanogenerators for Effective Treatment of *Helicobacter pylori* Infection without Disrupting Homeostasis of Intestinal Flora

Jiayin Yu, Zhihao Guo, Jiachang Yan, Changxin Bu, Chang Peng, Cuie Li, Rui Mao, Jian Zhang, Zhi Wang, Shi Chen, Meicun Yao, Zhiyong Xie, Chuan Yang, Yi Yan Yang, Peiyan Yuan* and Xin Ding**

Supporting Information

Gastric Acid-Responsive ROS Nanogenerators for Effective Treatment of Helicobacter pylori Infection without Disrupting Homeostasis of Intestinal Flora

Jiayin Yu, Zhihao Guo, Jiachang Yan, Changxin Bu, Chang Peng, Cuie Li, Rui Mao, Jian Zhang, Zhi Wang, Shi Chen, Meicun Yao, Zhiyong Xie, Chuan Yang, Yi Yan Yang, Peiyan Yuan*, and Xin Ding**

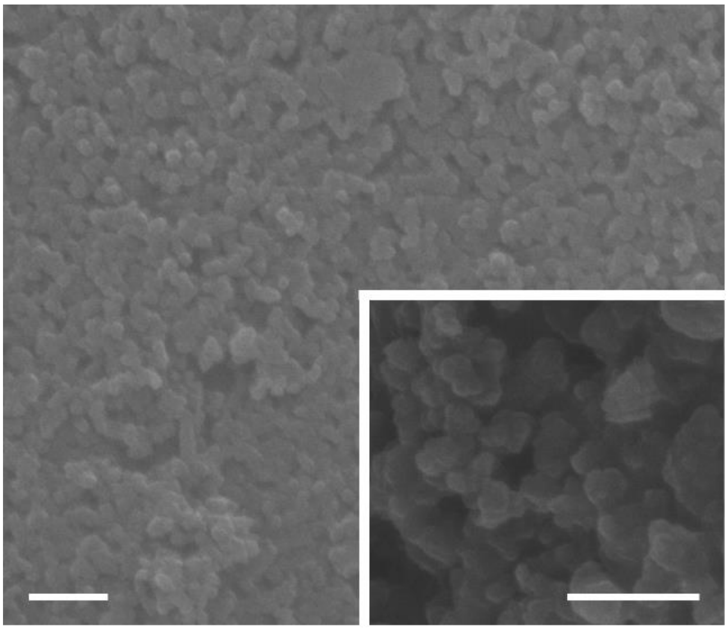

Figure S1. SEM image of Fe-HMME@DHA@MPN nanoparticles. The insert is the image at higher magnification obtained elsewhere of the same samples. Scale bar: 500 nm.

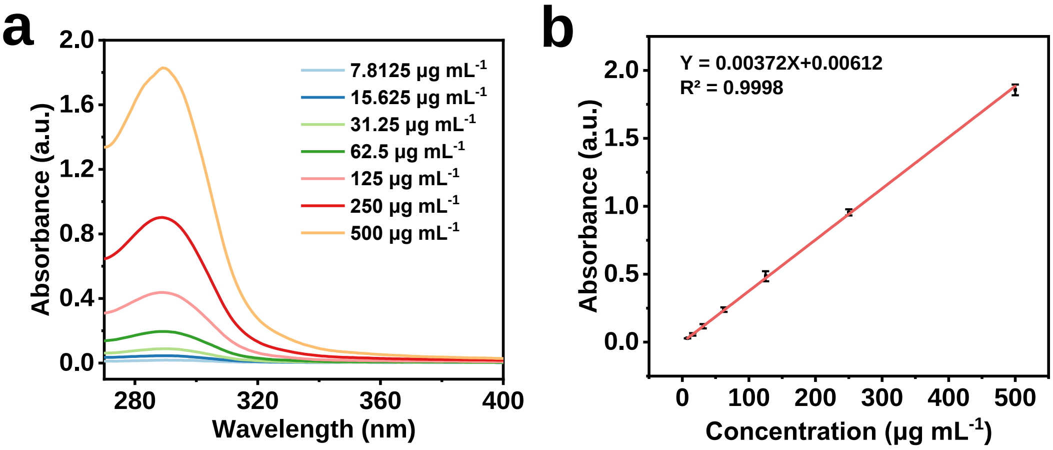

Figure S2. Quantification of DHA loading by using the UV-vis spectra. a) UV-vis absorption spectra of DHA stock solutions with different concentrations; b) The standard curve of DHA plotting in concentration versus absorbance values at 290 nm.

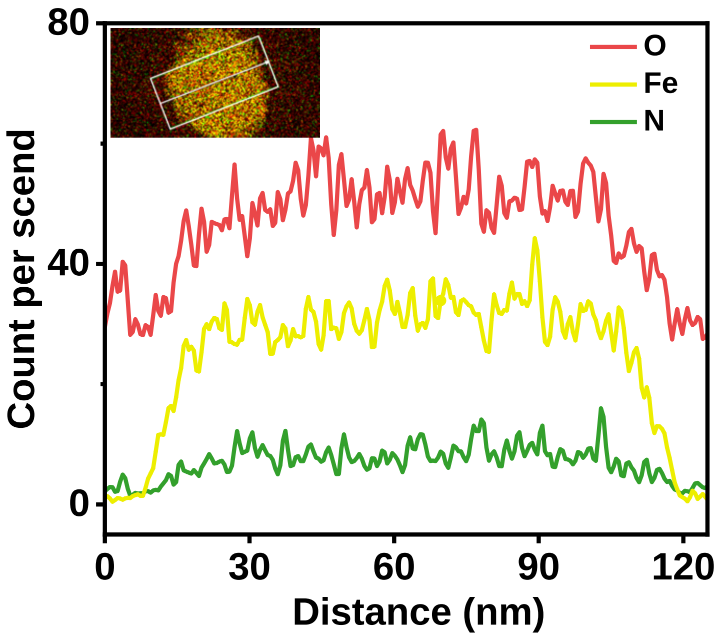

Figure S3. EDS line scan profiles of a single nanoparticle Fe-HMME@DHA@MPN for O, Fe and N analysis along the line shown in the superimposed STEM image (inset).

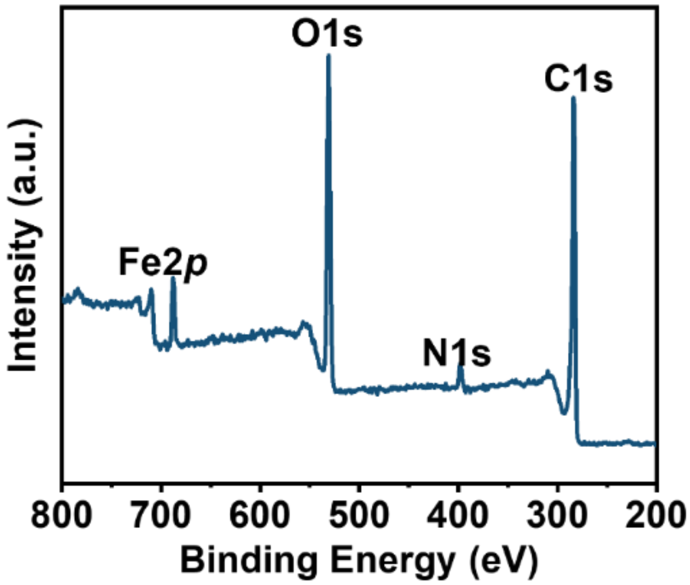

Figure S4. XPS survey spectrum of Fe-HMME@MPN nanoparticles.

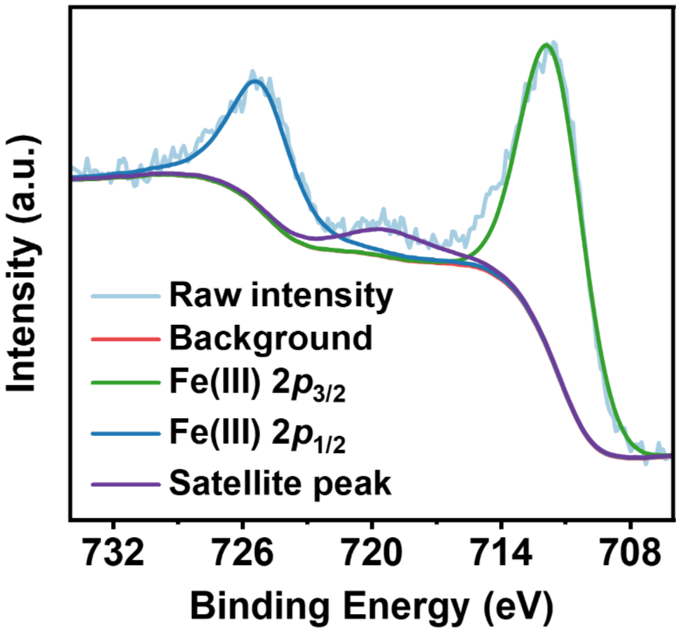

Figure S5. XPS survey spectrum of Fe 2p in Fe-HMME nanoparticles.

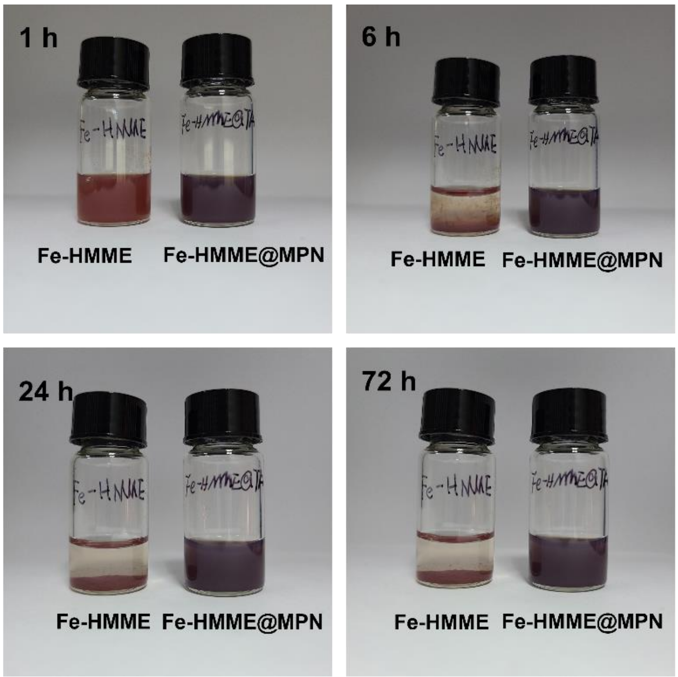

Figure S6. Photographs of Fe-HMME (left) and Fe-HMME@MPN (right) (100 μg mL^{-1}) dispersed in Milli-Q water for a different period of time (1 h, 6 h, 24 h and 72 h).

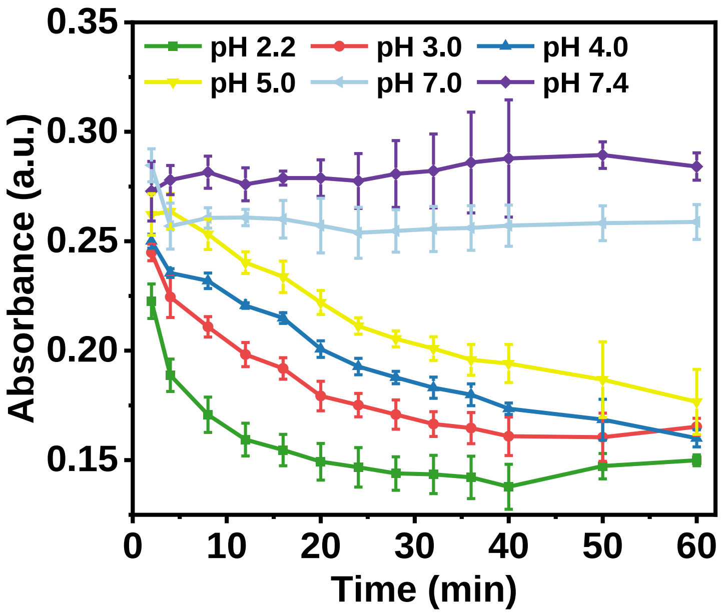

Figure S7. pH-responsive degradation kinetics of the Fe-HMME@DHA@MPN nanoparticles (1.0 mg mL^{-1}). The nanoparticles were stable in PBS (pH 7.4) and Milli-Q water (pH 7.0), while they degraded in SGF at lower pH. The lower the pH value, the faster the degradation is. (n = 3, data are presented as mean ± SD)

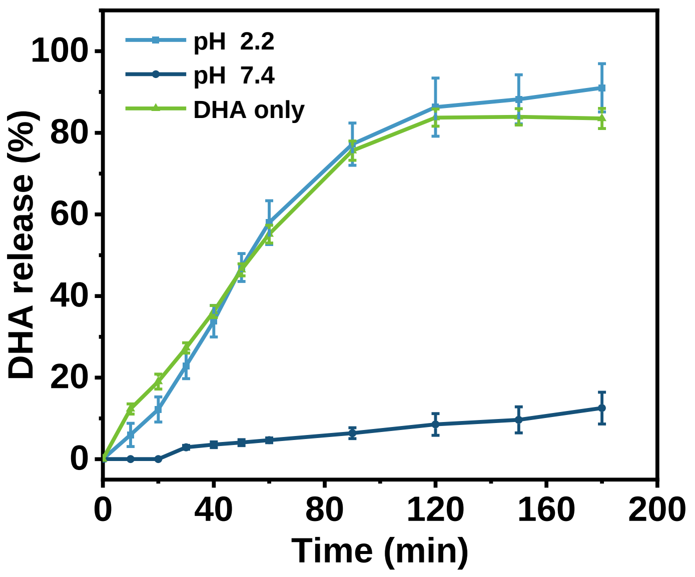

Figure S8. Release profiles of DHA from Fe-HMME@DHA@MPN nanoparticles (10 mg mL^{-1}) at different pH in comparison with free DHA. (n = 3, data are presented as mean ± SD)

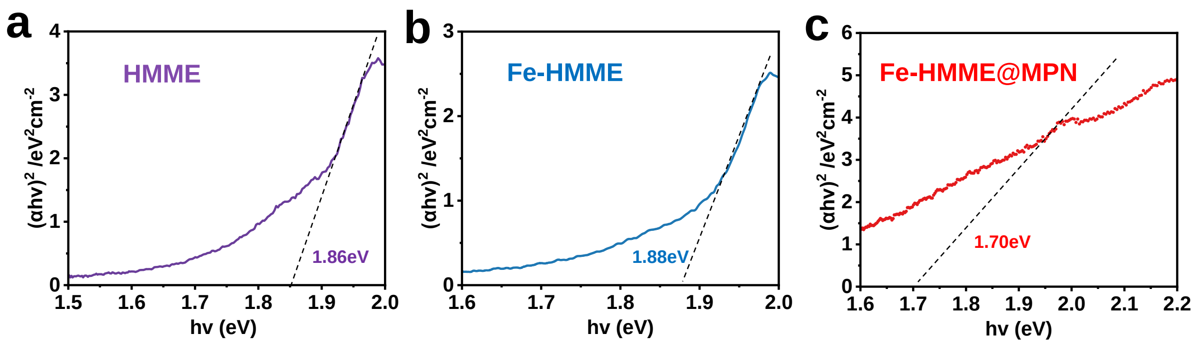

Figure S9. Optical bandgaps of a) HMME, b) Fe-HMME and c) Fe-HMME@MPN measured by UV-vis diffuse reflectance spectra.

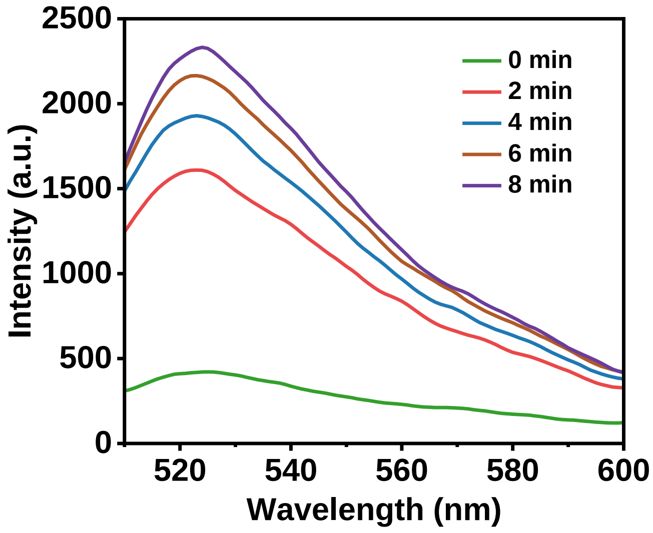

Figure S10. Fluorescence of molecular probe singlet oxygen sensor green (SOSG) solution containing Fe-HMME@MPN at 30 μg mL^{-1} treated with ultrasonication for different periods of time.

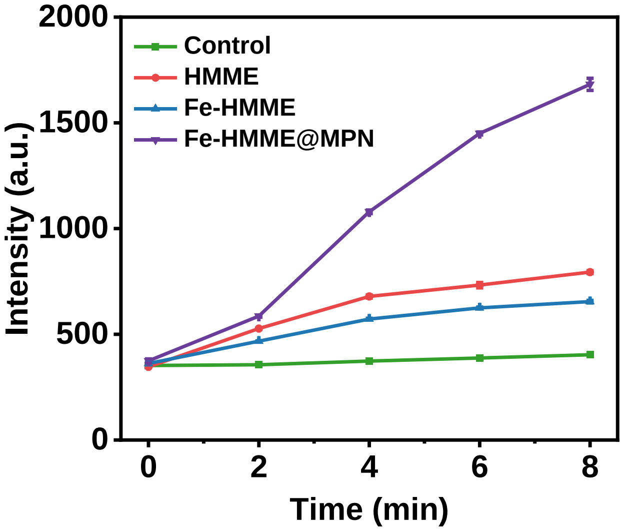

Figure S11. Singlet oxygen generation of HMME, Fe-HMME and Fe-HMME@MPN (30 μg mL^{-1}) characterized by the fluorescence of molecular probe singlet oxygen sensor green (SOSG) under the hypoxia condition. (The hypoxic condition was obtained by boiling in advance and injected with argon to remove dissolved oxygen in the solution.) (n = 3, data are presented as mean ± SD)

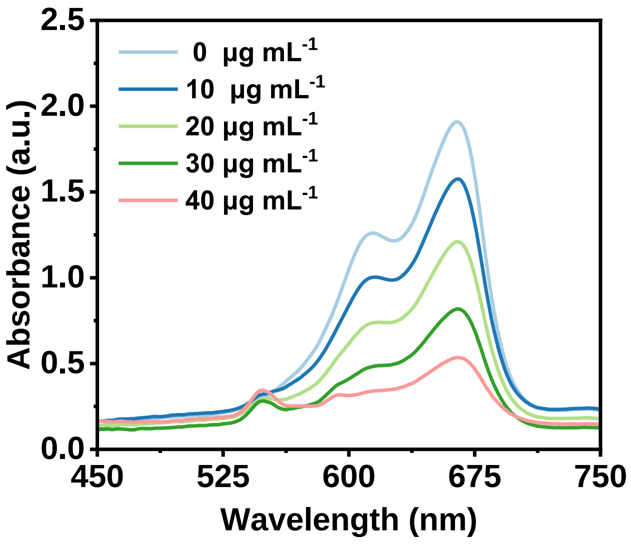

Figure S12. UV-vis absorption spectra of methylene blue, the indicator of ·OH production, incubated with Fe-HMME@MPN at various concentrations and H_2O_2 (1 mM) in the simulated gastric fluid (SGF, pH 2.2) for 30 min.

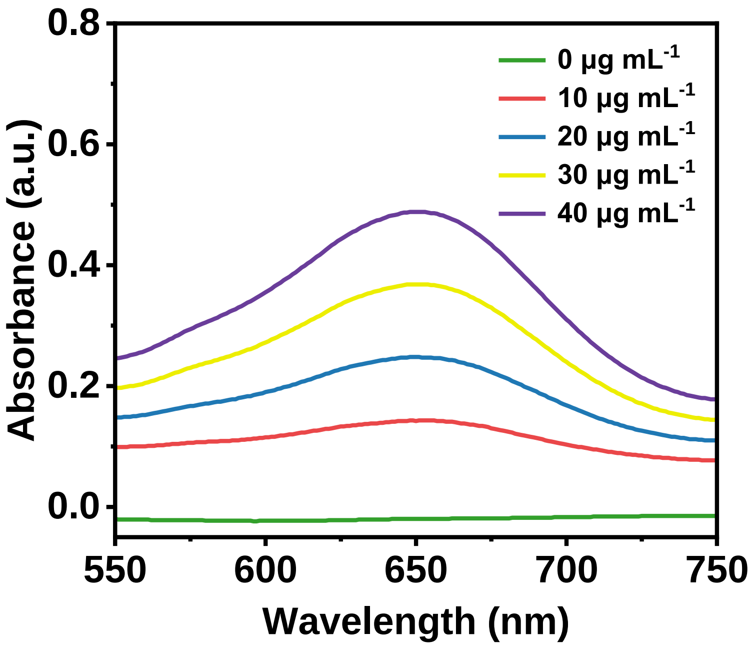

Figure S13. Concentration-dependent absorbance spectra of the peroxidase substrate 3,3',5,5'-tetramethylbenzidine (TMB) catalyzed by Fe-HMME@MPN nanoparticles in the presence of H_2O_2. (1 mM, incubate at 37°C for 10 min in pH 4.0 SGF)

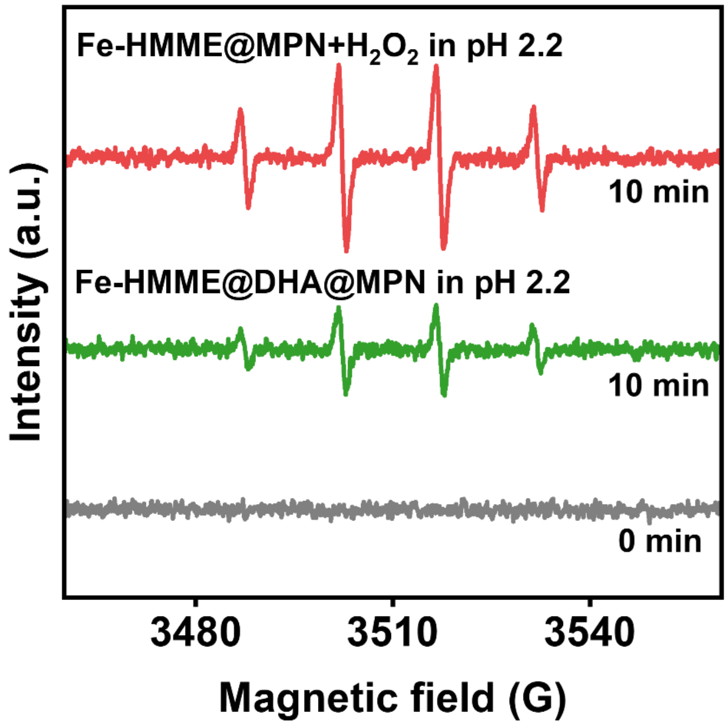

Figure S14. ESR spectra of Fe-HMME@MPN (90 μg mL^{-1} supplimented with 100 μM H_2O_2) and Fe-HMME@DHA@MPN (120 μg mL^{-1}) in pH 2.2 at 0 min and after 10 min, demonstrating ·OH generation.

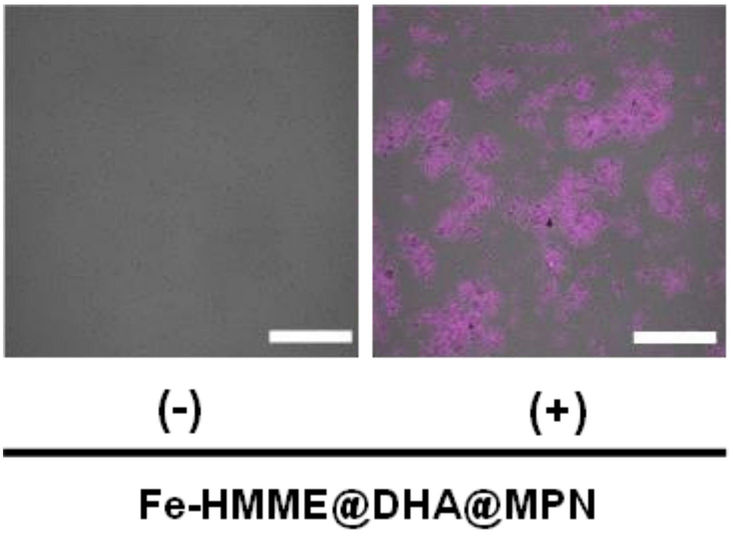

Figure S15. Confocal microscopic images of *H. pylori*. Aggregates were seen when Fe-HMME@DHA@MPN nanoparticles (purple emission from HMME) were incubated for 30 min with *H. pylori*. (scale bar: 50 μm).

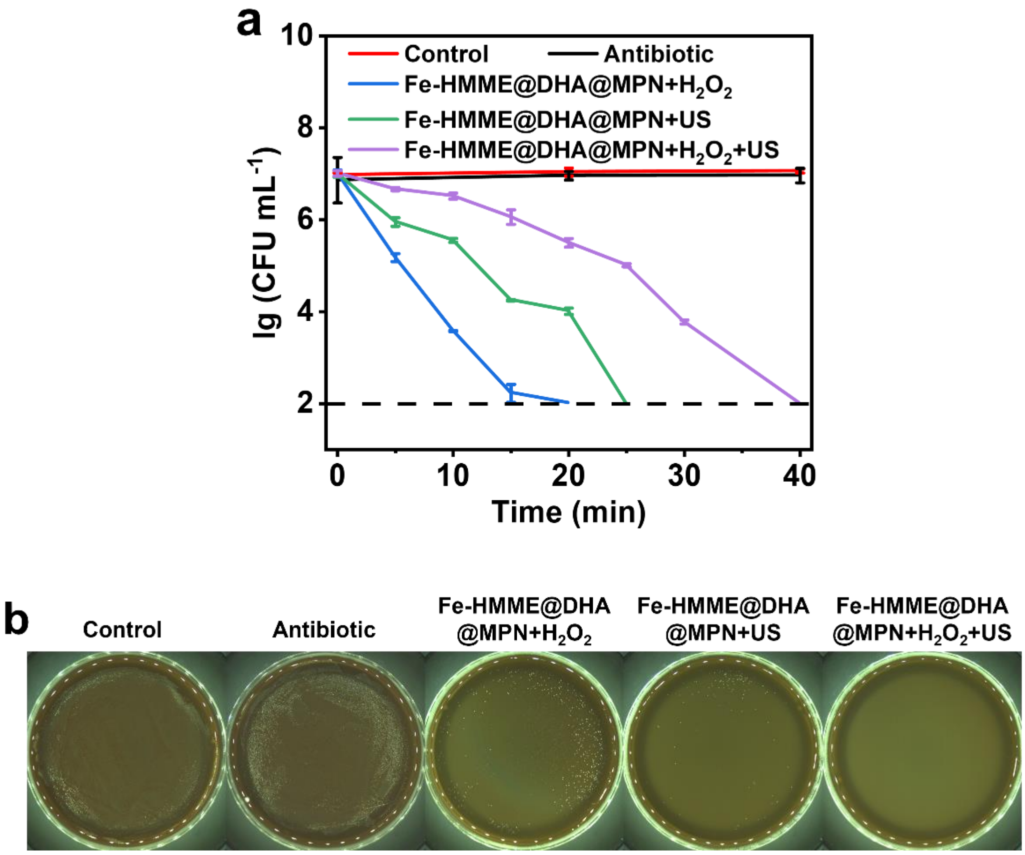

Figure S16. *In vitro* antibacterial activity of the catalytic ROS nanogenerator Fe-HMME@DHA@MPN against *H. Pylori*, in comparison with the antibiotic amoxicillin. a) Killing kinetics of Fe-HMME@DHA@MPN nanoparticles at 40 μg mL^{-1} and amoxicillin at 0.5 μg mL^{-1} against *H. pylori* (1×10^7 CFU mL^{-1}) under the different treatment conditions at pH 2.2. The dot line represents limit of detection (LOD). Ultrasonication for 2 min per 10 min incubation for a total of 40 min of incubation. (n = 3, data are presented as mean ± SD). b) Colony images of *H. pylori* after incubated with Fe-HMME@DHA@MPN nanoparticles (40 μg mL^{-1}) and amoxicillin (0.5 μg mL^{-1}) for 20 min in the simulated gastric fluid (pH 2.2).

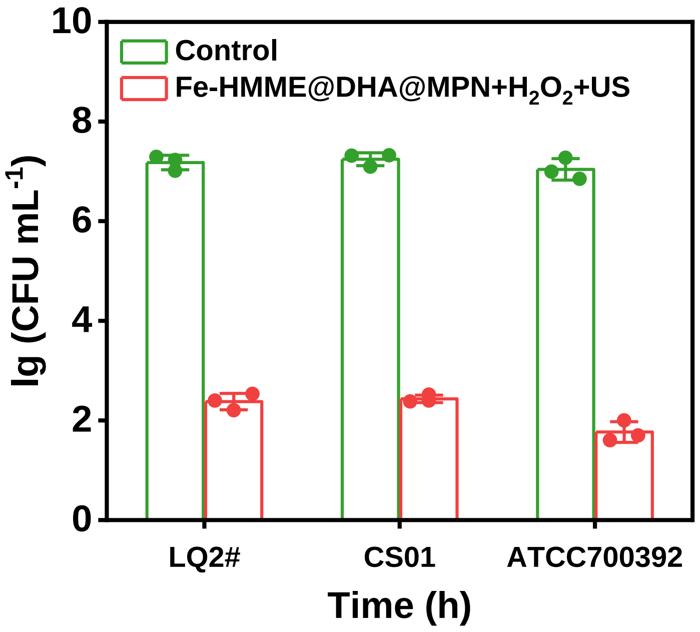

Figure S17. Colony counts of the other three *H. pylori* strains LQ2#, CS01, and ATCC 700392 (initial count: 1×10^7 CFU mL^{-1}) after incubated with Fe-HMME@DHA@MPN (40 μg mL^{-1}) for 20 min with US treatment 4 min in the simulated gastric fluid (pH 2.2). (n = 3, data are presented as mean ± SD)

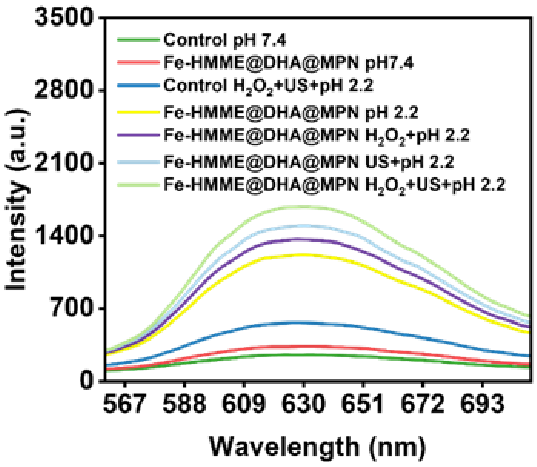

Figure S18. Fluorescence intensity of *H. pylori* incubated with the red dye PI that only stains the cells with damaged membrane. *H. pylori* (1×10^{7} CFU mL^{-1}) was treated with ROS nanogenerators Fe-HMME@DHA@MPN (40 μg mL^{-1}) under the different treatment conditions.

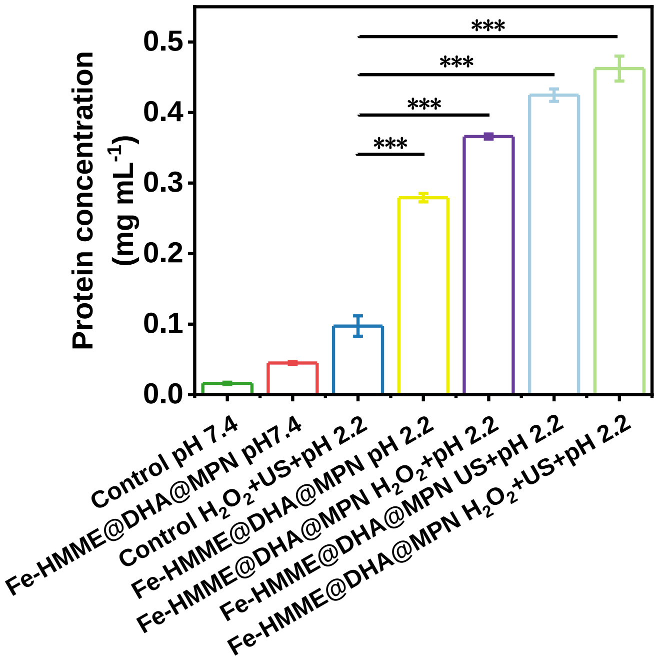

Figure S19. *H. pylori* protein leakage measured by BCA assay. *H. pylori* (1×10^{7} CFU mL^{-1}) was treated with ROS nanogenerators Fe-HMME@DHA@MPN (40 μg mL^{-1}) under the different treatment conditions. (n = 3, data are shown as means ± SD, and the significant difference was analyzed by One-way ANOVA with Tukey's post hoc test. $*p < 0.05$, $**p < 0.01$, $***p < 0.001$ and ns representing non-significance.)

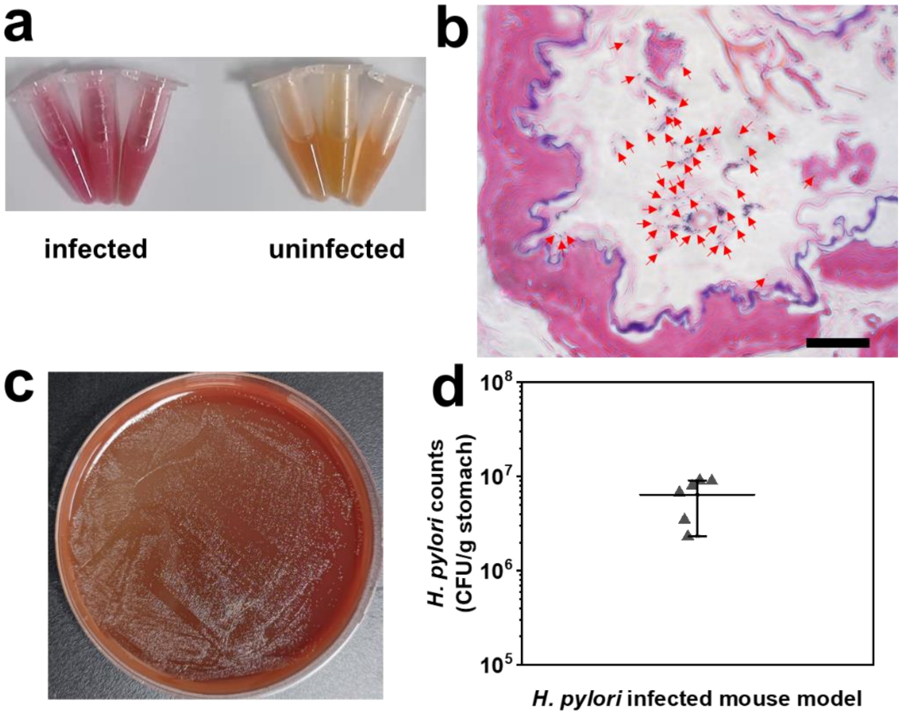

Figure S20. Establishment of *H. pylori*-infected mouse model. a) Urease test performed after gavage with *H. pylori* susupention (1×10^8 CFU mL^{-1}) once a day for 4 consecutive days. b) Gram staining of a slice from the gastric mucosa of *H. pylori*-infected mouse. Red arrows point to *H. pylori*. Scale bar: 50 µm. c) Representitive photograph of *H. pylori* colonies after gastric mucosa reculture. d) Quantification of bacterial burden in the stomach of *H. pylori*-infected mice. (n = 6, data are shown as means ± SD)

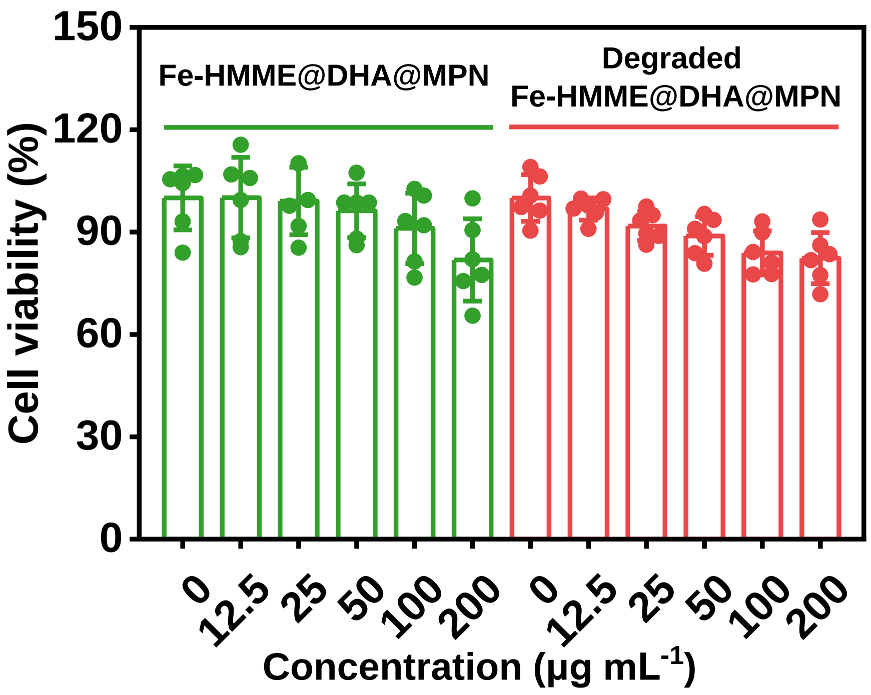

Figure S21. Viability of GES-1 cells after treatment with Fe-HMME@DHA@MPN or their degradation products at different concentrations for 24 h. (n = 6, data are presented as means ± SD)

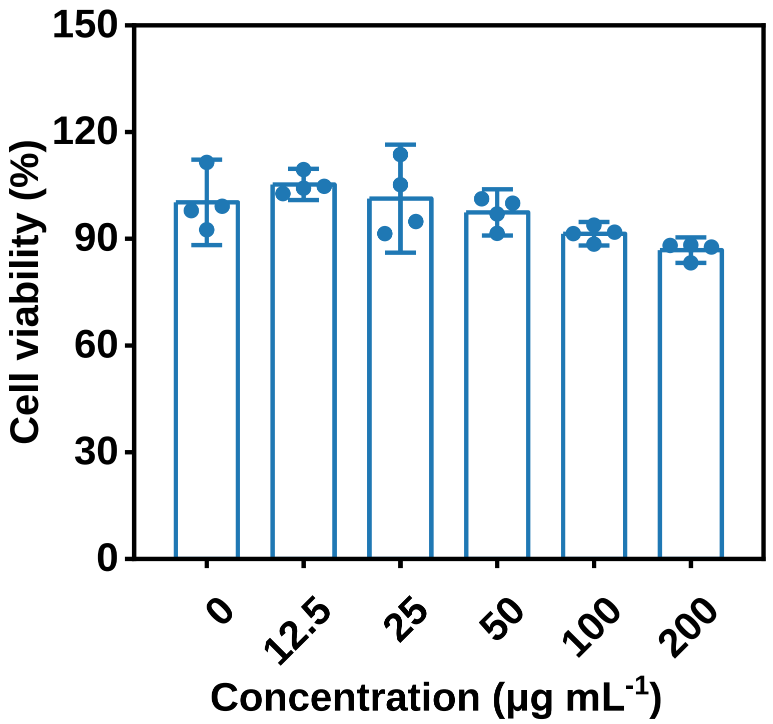

Figure S22. Viability of HUVEC cells after treatment with Fe-HMME@DHA@MPN nanoparticles at different concentrations for 24 h. (n = 4, data are presented as means ± SD)

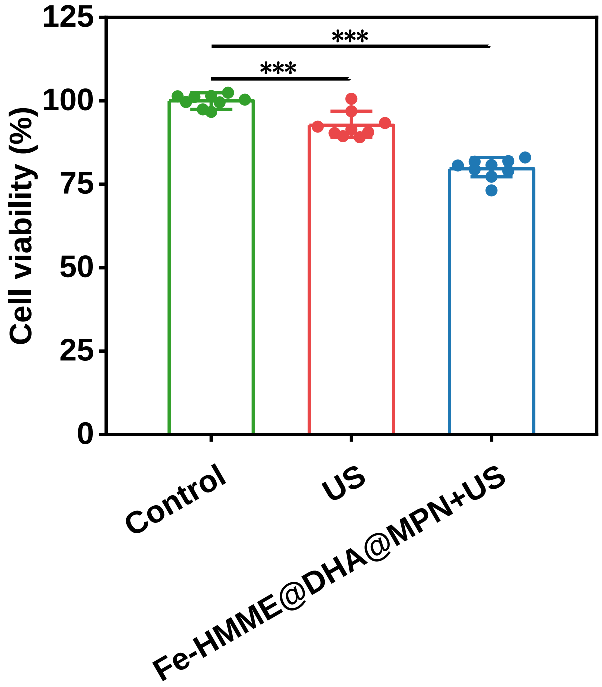

Figure S23. Viability of GES-1 cells. GES-1 cells were incubated with Fe-HMME@DHA@MPN (40 μg mL-1) and treated with US twice (2 min each time), then continued to incubate for 24 h. (n = 9, data are presented as means ± SD, and the significant difference was analyzed by One-way ANOVA with Tukey's post hoc test. *p < 0.05, **p < 0.01, ***p < 0.001 and ns representing non-significance.)

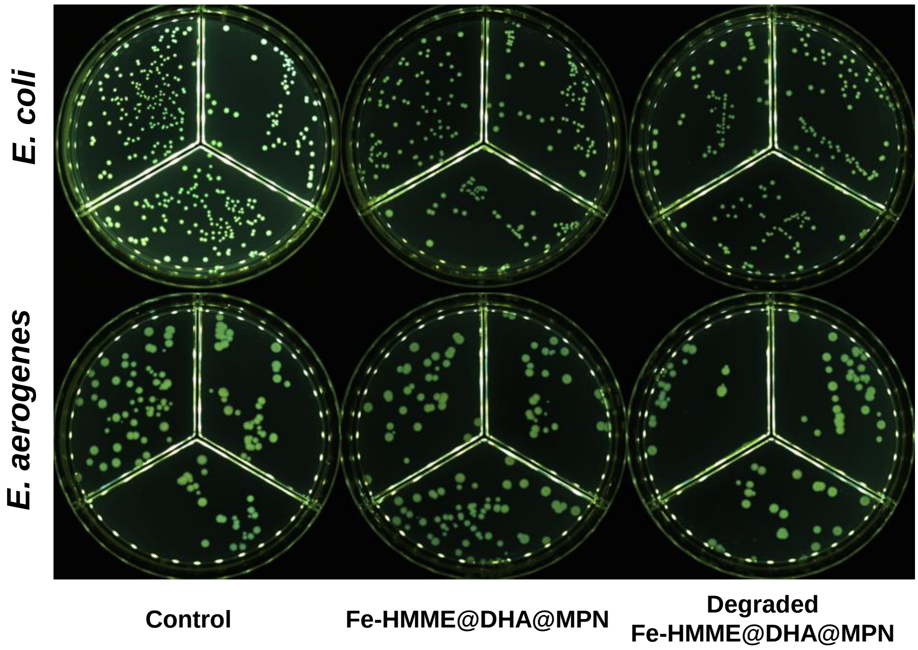

Figure S24. Representative colony photographs of *E. coli* and *E. aerogenes* (1×10^8 CFU mL^{-1}) with or without treatment of Fe-HMME@DHA@MPN nanoparticles or their degraded products (100 μg mL^{-1}) for 6 h.

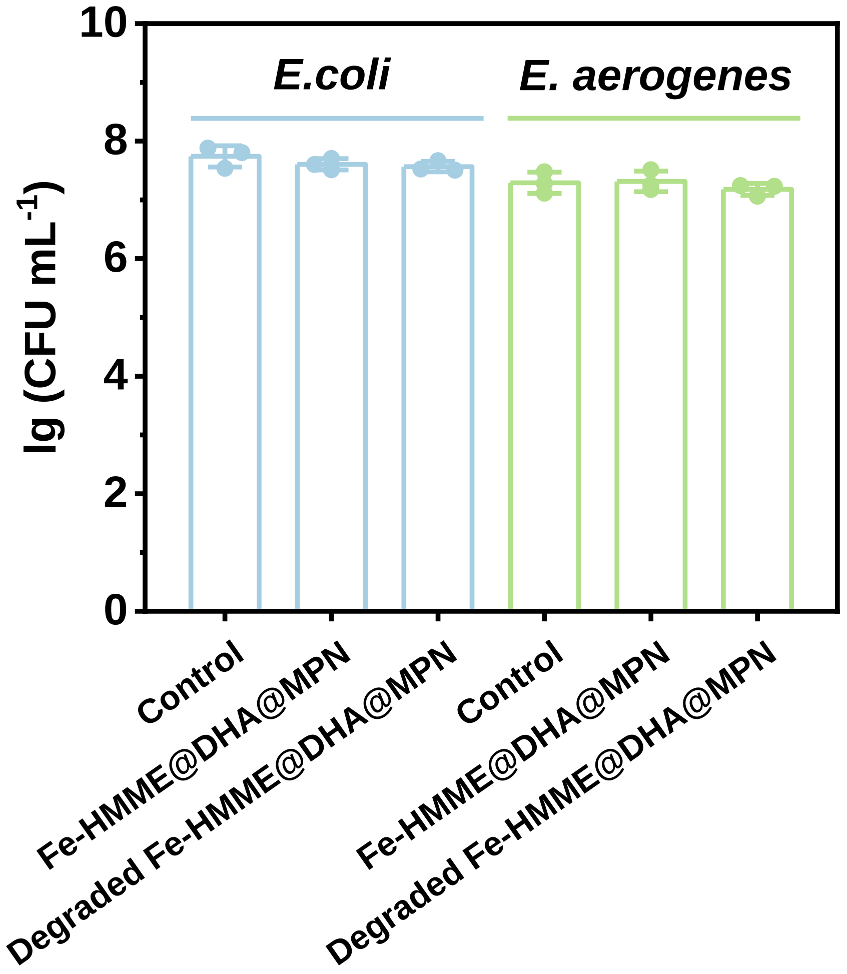

Figure S25. Colony quantification of *E. coli* and *E. aerogenes* (1×10^8 CFU mL^{-1}) with or without treatment of Fe-HMME@DHA@MPN nanoparticles or their degraded products (100 μg mL^{-1}) for 6 h. (n = 3, data are presented as mean ± SD)

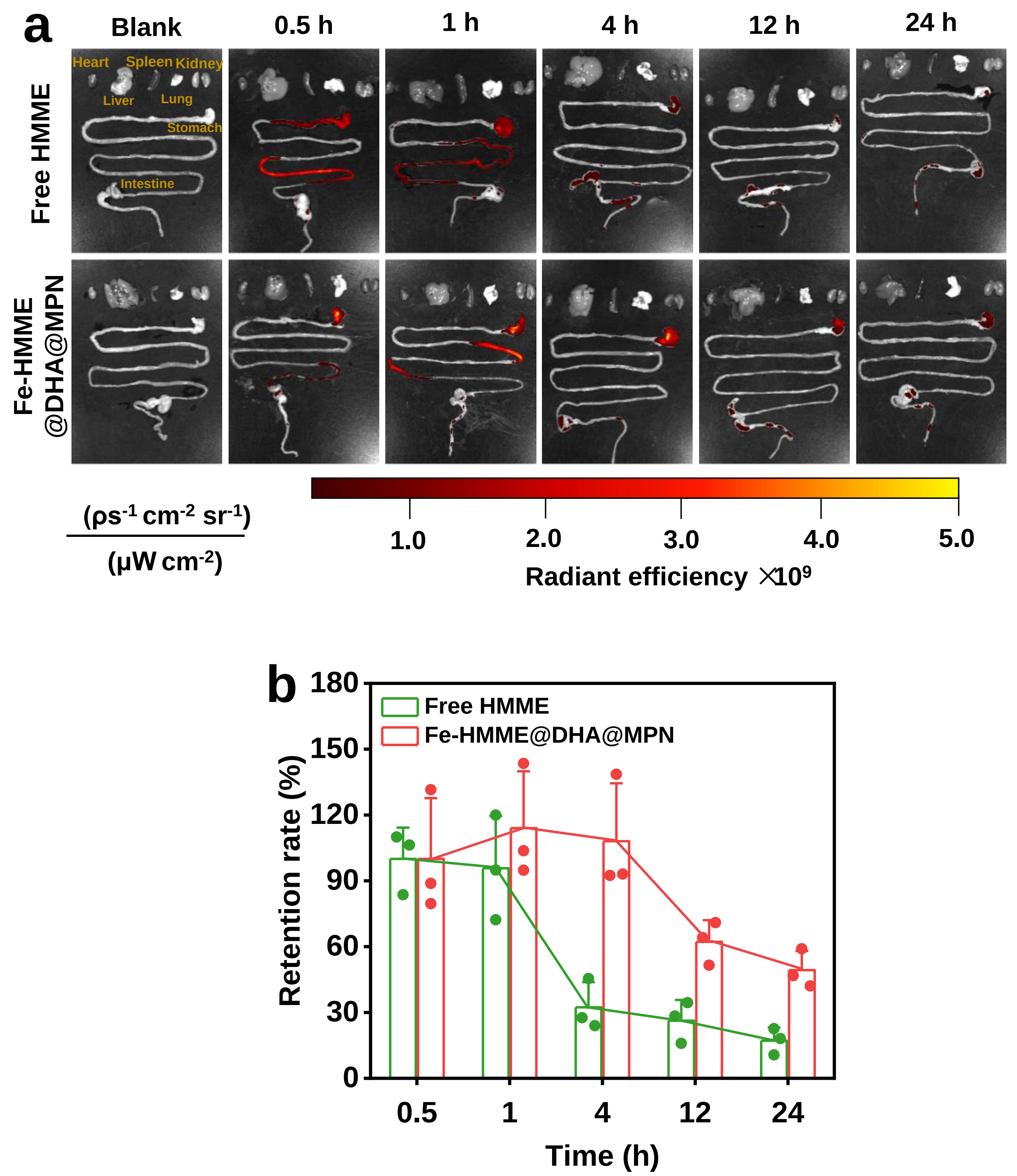

Figure S26. *In vivo* biodistribution of free HMME and Fe-HMME@DHA@MPN nanoparticles after intragastic adiministration. a) Representative *ex vivo* fluorescence imaging of major organs (heart, liver, spleen, lung, kidney, stomach, and intestine) from BALB/c mice treated with free HMME (15 mg kg^{-1}) or Fe-HMME@DHA@MPN (30 mg kg^{-1}). The major organs were harvested and imaged at 0.5, 1, 4 and 24 h post administration. The intrinsic fluorescence of HMME was observed at $\lambda_{Ex/Em}$ = 620/670 nm. b) Relative retention ratio of free HMME and Fe-HMME@DHA@MPN in stomach harvested from the mice receiving the treatment in a). *Ex vivo* images were quantified by measuring fluorescence intensity at the stomach. All values were expressed as means ± SD (n = 3).

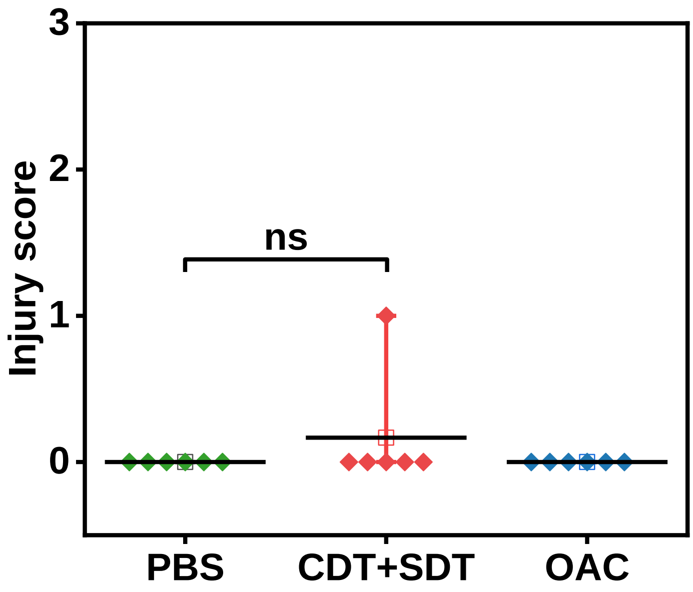

Figure S27. Injury score of gastric mucosa obtained by analyzing the H&E staining images in Figure 5f in maintext. (n = 6, data are presented as means ± SD, and the significant difference was analyzed by Student's t-test. *p < 0.05, **p < 0.01, ***p < 0.001 and ns representing non-significance.)

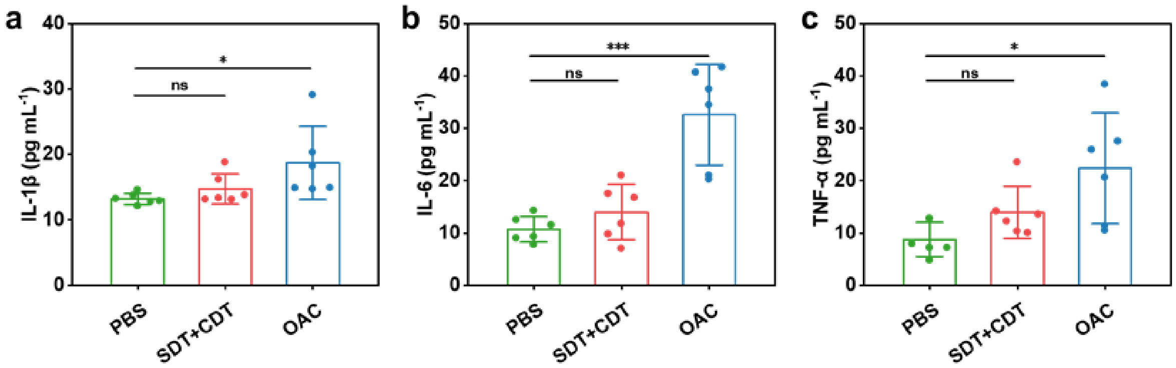

Figure S28. Level of proinflammatory cytokines IL-1β, IL-6, and TNF-α in gastric homogenate of mice under the different treatment conditions, determined by enzyme-linked immunosorbent assay (ELISA). (n = 6, data are presented as means ± SD, and the significant difference was analyzed by One-way ANOVA with Tukey's post hoc test. *p < 0.05, **p < 0.01, ***p < 0.001 and ns representing non-significance.)

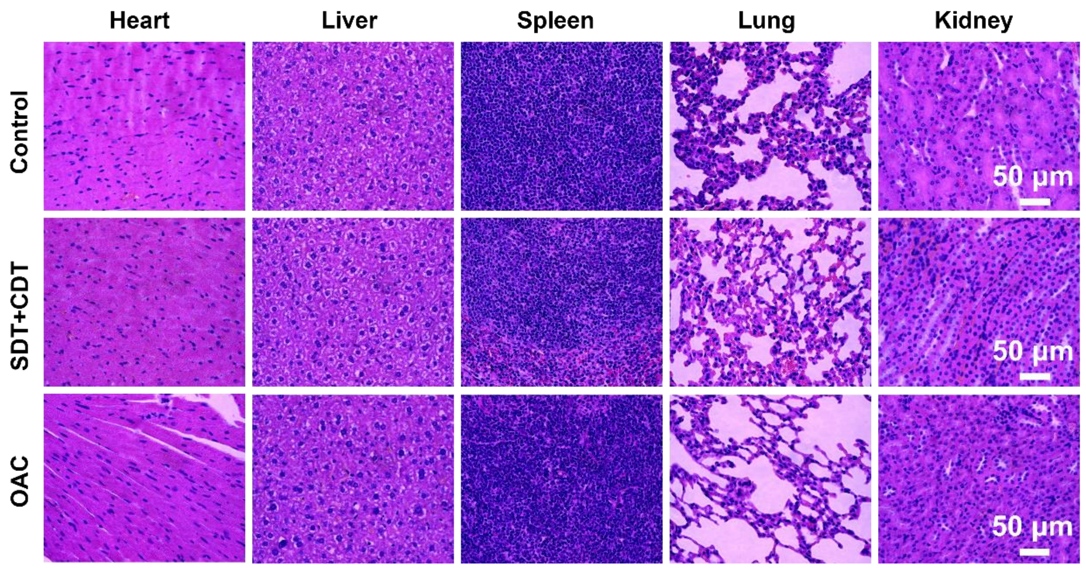

Figure S29. H&E staining images of heart, liver, spleen, lung and kidney of mice after the specified treatments (Control: 100 μL phosphate buffer saline, CDT+SDT: 30 mg kg^{-1} of Fe-HMME@DHA@MPN + US, OAC: combination of 400 μmol kg^{-1} of omeprazole, 28.5 mg kg^{-1} of amoxicillin and 14.3 mg kg^{-1} of clarithromycin).

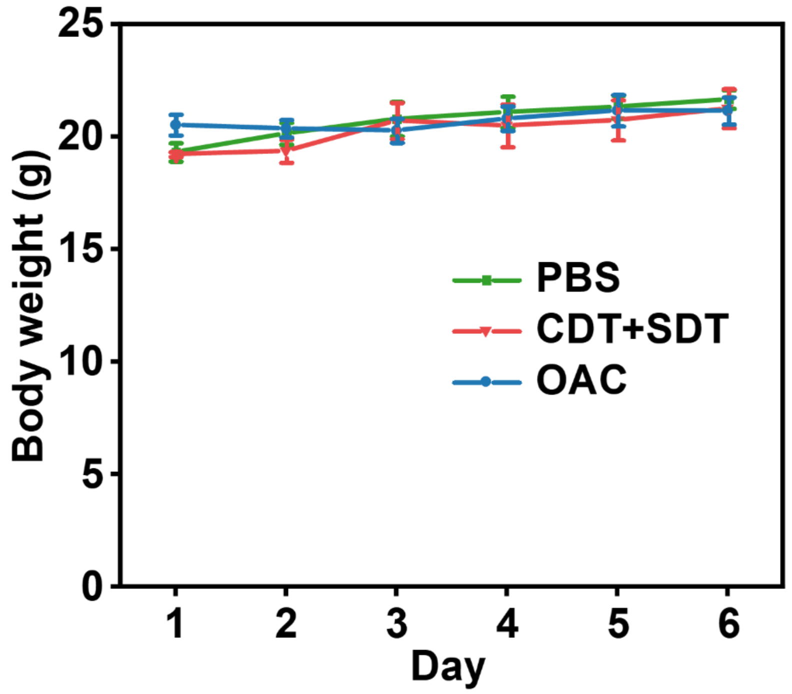

Figure S30. Body weight of the mice following the treatment with PBS, CDT+SDT or OAC therapy (Control: 100 μL phosphate buffer saline, CDT+SDT: 30 mg kg^{-1} of Fe-HMME@DHA@MPN + US, OAC: combination of 400 μmol kg^{-1} of omeprazole, 28.5 mg kg^{-1} of amoxicillin and 14.3 mg kg^{-1} of clarithromycin). (n = 6, data are presented as mean ± SD)

Table S1. Antibacterial activity of Fe-HMME@DHA@MPN against *H. pylori*.[a)]

	Strains	Drug sensitivity [c)]	MBC ($\mu g\ mL^{-1}$)
Fe-HMME@DHA@MPN in normal saline	ATCC 43504	R(MTZ)	>128
Fe-HMME@DHA@MPN + 200 μM H_2O_2 in SGF	ATCC 43504	R(MTZ)	40
Fe-HMME@DHA@MPN + US [b)] in SGF	ATCC 43504	R(MTZ)	10
Fe-HMME@DHA@MPN +200 μM H_2O_2 + US in SGF	ATCC 43504	R(MTZ)	5
Fe-HMME@DHA@MPN +200 μM H_2O_2 + US in SGF	ATCC 700392	S	5
Fe-HMME@DHA@MPN +200 μM H_2O_2 + US in SGF	CS01	R(CLR)	10
Fe-HMME@DHA@MPN +200 μM H_2O_2 + US in SGF	LQ2#	R(CLR, AMO, LEF)	5

[a)] The samples and bacteria were incubated for 30 min in SGF containing 10 mM fresh urea at pH 2.2, and the bacterial suspension was then placed and streaked on the Columbia agar plate, followed by culture for 3-4 days before counting the number of the bacteria colonies.

[b)] US: The mixture was exposed to ultrasound (1.0 MHz, 70% duty cycle, 1.5 W cm^{-2}) during the incubation (2 min each time, 3 times during 30 min).

[c)] S: drug suseptible, R: drug resistant, MTZ: metronidazole, CLR: clarithromycin, AMO: amoxicillin, LEF: levofloxacin.

Table S2. Serum biochemistry analysis of mice with intragastric administration of Fe-HMME@DHA@TA nanoparticles or the antibiotic-based triple therapy OAC.

Treatment	ALT (U/L)	AST (U/L)	Urea (mM)	Creatinine (μM)	Sodium ion (mM)	Potassium ion (mM)	Iron ion (μM)
PBS	37.1 ± 5.7	122.0±21.0	7.7±0.7	24.7±2.7	154.0±2.7	3.8±0.3	18.3±1.1
Fe-HMME @DHA @TA	40.1±3.6 (*P=0.49*)	121.6±13.3 (*P=0.98*)	9.5±1.7 (*P=0.18*)	24.9±3.0 (*P=0.95*)	153.2±1.1 (*P=0.66*)	4.1±0.2 (*P=0.19*)	18.6±1.4 (*P=0.82*)
OAC	54.9±8.0 (*P=0.03*)	161.7±10.7 (*P=0.04*)	7.6±1.2 (*P=0.93*)	27.1±3.3 (*P=0.39*)	155.7±1.1 (*P=0.36*)	4.2±0.4 (*P=0.20*)	17.5±1.2 (*P=0.41*)

* PBS and OAC treatments were served as control groups. Data are presented as mean ± SD (n = 3) and analyzed by student t-test as compared with PBS group. $P \leq 0.05$ indicates statistical significance.

Table S3. Sequences of the primers used for quantitative real-time PCR.

**Primer name**	**Primer sequence**	**Note**
Bac16s8F	5-AGAGAGTTTGATCCTGGCTCAG-3	Forward primer
Bac16s338R	5-TGCTGCCTCCCGTAGGAGT-3	Reverse primer
